# Isolation of highly copper-resistant bacteria from deep-sea hydrothermal fields and description of a novel species *Marinobacter metalliresistant* sp. nov

**DOI:** 10.3389/fmicb.2024.1390451

**Published:** 2024-08-21

**Authors:** Tong Yu, Meng Qin, Zongze Shao, Yuemei Zhao, Xiang Zeng

**Affiliations:** ^1^Key Laboratory of Marine Biogenetic Resources, Third Institute of Oceanography, Ministry of Natural Resources, Xiamen, China; ^2^School of Marine Sciences, China University of Geosciences, Beijing, China; ^3^Faculty of Marine Biology, Xiamen Ocean Vocational College, Xiamen, China

**Keywords:** copper-resistant bacteria, deep-sea hydrothermal fields, Indian Ocean, extracellular polymeric substances, *Marinobacter metalliresistant* sp. nov

## Abstract

**Introduction:**

Hydrothermal vents, rich in heavy metals, provided a unique niche for heavy metal resistant microbes. However, knowledge about copper resistant microbes in deep sea hydrothermal vents is still limited.

**Methods:**

The copper-resistant bacteria were isolated from deep-sea hydrothermal vent samples and conducted thorough physical, phylogenetic, and genomic analyses to elucidate their copper resistance capability and related genes.

**Results:**

Twelve highly copper-resistant bacteria (up to 6-10 mM) were isolated from deep sea hydrothermal fields They were affiliated with the *Pseudoalteromonas* (4), *Marinobacter* (3), *Halomonas* (2), *Psychrobacter* (1), and *Pseudomonas* (1) genus in the α-Proteobacteria, and the *Sphingomonas* (1) genus in the β-Proteobacteria. The presence of copper in the medium obviously induced the amount of polysaccharides and proteins in the crude extracellular polymeric substances (EPS) produced by *Halomonas* sp. CuT 3-1, *Pseudoalteromonas* sp. CuT 4-3 and *Marinobacter metalliresistant* CuT 6, which could absorb 40 to 50 mg•g^−1^ copper. We further described a novel species, *Marinobacter metalliresistant* sp. nov. CuT 6^T^, which exhibited a higher copper resistance and encoded more heavy metal resistance-related genes than other Marinobacter species.

**Discussion:**

It revealed that the copper resistance capability exhibited by these strains in hydrothermal fields is likely attributed to the production of exopolymeric substances, such as polysaccharides and proteins, as well as active transport or efflux mechanisms for heavy metals.

## 1 Introduction

Deep-sea hydrothermal fields are a source of heavy metals such as Cu, Zn, V, and U (Hu et al., 2022). For example, hydrothermal fluids continued to contribute a significant proportion (5–14%) of the Earth’s oceanic copper inventory in the form of organically bound complexes, including metallic mineral deposits (Zierenberg et al., 2000; Sander and Koschinsky, 2011; Stüeken, 2020). The content of copper in deep-sea hydrothermal vents has been documented to reach notably high levels, 141.15–551.58 mg/kg in the hydrothermal sediments (Zhu and Zhou, 2021) and 9.6–228.0 g/kg in the hydrothermal sulfides (Bonatti et al., 1979; Dekov et al., 2010; Sander and Koschinsky, 2011; Sun et al., 2011; Liao et al., 2018). These values are notably higher than the average crustal abundance of copper (63 mg/kg), highlighting the remarkable potential of these vents for copper-resistant bacteria.

Heavy metal resistance and acquisition are important adaptive strategies in bacteria in terrestrial and aquatic environments, but their distribution and mechanisms were unexplored (Mathivanan et al., 2021). Most metallophiles were found in the metal processing industries or dumping sites, not natural habitats such as deep-sea or terrestrial hydrothermal sources (Dopson and Holmes, 2014; Kanekar and Kanekar, 2022). The reported neutrophilic copper-resistant microorganisms predominantly belong to diverse bacterial phyla, including α-proteobacteria (*Methylobacterium* sp.), β-proteobacteria (*Alcaligenes* sp.), γ-proteobacteria (*Pseudomonas* sp., *Salmonella* sp., and *Acinetobacter* sp.), Firmicutes (*Bacillus* sp. and *Streptococcus* sp.), and fungi (*Paecilomyces lilacinus*, *Aspergillus terreus*, and *Cladosporium* sp.). Furthermore, some mineral-oxidizing hyperthermophilic archaea such as *Acidianus brierleyi*, *Sulfolobus metallicus,* and *Metallosphaera sedula*, which were from terrestrial hot springs, exhibited remarkable tolerance to elevated copper concentrations (Harrison, 2016). Among them, *Bacillus* sp. recorded the highest copper-resistant ability up to 5.5 mM (Kunito et al., 1997). However, little attention has been paid to heavy metal-related microorganisms in deep-sea hydrothermal fields before. The bacterial strain *Alcanivorax* sp. VBW004 from a shallow hydrothermal vent has demonstrated remarkable copper resistance capabilities, tolerating concentrations up to 2.4 mM. This is attributed to the presence of copper resistance genes, such as *copB* (Ramasamy et al., 2020). Vetriani et al. reported these bacteria including *Alcanivorax*, *Bacillus*, *Pseudomonas*, *Marinobacter*, *Pseudoalteromonas*, and *Halomonas* had mercury resistance ability and an ecological role in mercury detoxification in the vent environment (Vetriani et al., 2005). Microorganisms inhabiting hydrothermal vents exhibit a tolerance to heavy metals, yet most of them are unable to thrive in environments with elevated concentrations. For example, *Nitratiruptor* sp. SB155-2 (belonging to phylum Campylobacterota) from deep-sea hydrothermal vents could only grow under Cu (II) stress below 0.1 mM copper through high-affinity efflux systems (Ares et al., 2022). In this study, we first isolated several high copper-resistant bacteria from deep-sea hydrothermal fields in the South West Indian Ocean ridge. These strains could tolerate concentrations of up to 6–10 mM Cu (II) and could grow under the stress of higher than 2 mM Cu (II). Their potential resistant mechanism based on their genome analysis was discussed to understand their adaptation in deep-sea hydrothermal vents.

## 2 Materials and methods

### 2.1 Sample collection

The samples were collected using a TV grab sampler at several sites at a depth of 1,453–2,653 m in hydrothermal fields of the southwest Indian Ocean in February 2020 during the cruise of DaYang Hao (Table S1). The sediments, sulfides, and oxides were fractionated on a clean bench and subsample was further used to inoculate (1/10, w/v) a sterile liquid enrichment media at 25°C on board. The sulfides were composed primarily of hydrothermal chimney fragments, predominantly consisting of pyrite (FeS₂), chalcopyrite (CuFeS₂), and sphalerite (ZnS), among others. The oxide samples were dominated by oxides/hydroxides of Fe, Mn, and Si. Furthermore, the sediments were composed of calcareous ooze, with traces of sulfides, exhibiting a significant proportion of calcium carbonate, reaching up to 50% in volume.

### 2.2 Enrichment and isolation of copper-resistant bacteria

Enrichment of copper-resistant bacteria was conducted in 100 mL of marine broth (MB) with 6 mM Cu (II) as copper sulfate (CuSO_4_•5H_2_O) in 250 mL Erlenmeyer flasks. Per 1,000 mL of MB (Becton, Dickinson) contained 5.0 g peptone, 1.0 g yeast extract, 0.1 g ferric citrate, 19.45 g NaCl, 5.9 g MgCl_2_, 1.8 g CaCl_2_, 0.55 g KCl, 0.16 g NaHCO_3_, 34.0 mg SrCl_2_, 22.0 mg H_3_BO_3_, 0.08 g KBr, 4.0 mg Na_2_SiO_3_, 2.4 mg NaF, 1.6 mg NH_4_NO_3_, 8.0 mg Na_2_HPO_4_, 3.24 g MgSO_4_, and the solution was adjusted to pH 7.0. The enrichment culture was incubated at 28°C for 3–5 days on board and stored with glycerol at −20°C. In the lab, the subculture was incubated at 28°C for 24 h, with shaking (150 rpm). Cu (II)-resistant bacterial strains were purified by repeatedly streaking them on marine broth agar (MA) plates containing 6 mM Cu (II). These isolates were deposited in the Marine Culture Collection of China, MCCC^1^ (Table 1).

**Table 1:** Isolates with high copper-resistant activity in this study.

Strains	MCCC deposition no.	Source	Closest species (Identity %)	16 s rDNA accession no.	Maximum copper-resistant concentrations (mM)	EPS production
CuT 1–1	M25614	Sediments	*Stutzerimonas frequens* DNSP21 (99.86%)	OR632623	9	**+**
CuT 1–2	M25615	Sediments	*Marinobacter vinifirmus* FB1 (99.71%)	OR632624	6	**+**
CuT 2–2	M25659	Oxides	*Pseudoalteromonas agarivorans* DSM 14585 (99.57%)	OR632645	9	**+**
CuT 3–1	M25617	Sediments	*Halomonas meridiana* DSM 5425 (99.85%)	OR632643	10	**+**
CuT 3–2	M25616	Sediments	*Pseudoalteromonas agarivorans* DSM 14585 (99.93%)	OR632626	6	**−**
CuT 4–1	M25618	Sulfides	*Marinobacter guineae* M3B (99.20%)	OR632628	6	**+**
CuT 4–2	M25619	Sulfides	*Halomonas meridian* DSM 5425 (99.78%)	OR632629	10	**+**
CuT 4–3	M25620	Sulfides	*Pseudoalteromonas agarivorans* DSM 14585 (99.71%)	OR632630	10	**+**
CuT 5	M25621	Sediments	*Pseudoalteromonas agarivorans* DSM 14585 (99.93%)	OR632631	10	**+**
CuT 6	M25622	Oxides	*Marinobacter guineae* M3B (97.46%)	OR632632	8	**+**
CuT 7	−	Sediments	*Sphingomonas dokdonensis* DS-4 (99.55%)	OR632633	6	**+**
CuT 10	M25613	Sediments	*Psychrobacter oceani* 4 k5 (99.3%)	OR632622	7	**+**

### 2.3 Identification and phylogenetic analysis of isolates

DNA of each isolate was extracted by the Genomic DNA extraction kit (SBS Genetech Co., Ltd., Shanghai). Two universal primers corresponding to *E. coli* positions 27F (5’-AGATTTGATCMTG GCTCAG-3′) and 1492R (5’-TACGGYTACCTTGTTACGACTT-3′) were used for PCR amplification of the 16S ribosomal RNA of the isolates, and sequences information were obtained through Sanger sequencing. EzBioCloud^2^ was used for homology searches on 16 s rDNA genes (Yoon et al., 2017a). Phylogenetic analysis was performed using MEGA X (64-bit) (Larkin et al., 2007) after multiple alignments of the sequence data with Clustal W. Distances were calculated using distance options according to Kimura’s two-parameter model and clustering was performed using the neighbor-joining (NJ) methods. Sequences of related taxa were obtained from the GenBank database and supported with bootstrap values based on 1,000 replications.

### 2.4 Detection and identification of EPS production

To identify potential EPS production by bacterial isolates, the Congo red agar (CRA) plate test was utilized, which contained 5.0 g/L peptone, 1.0 g/L yeast extract, 30.0 g/L NaCl, and 15.0 g/L agar, with pH adjusted to 7.0. After autoclaving (121°C, 15 min), 10.0 g/L of glucose and 0.8 g/L of Congo red indicator were added (Maalej et al., 2015). The purified strains were inoculated onto a CRA plate and incubated at 28°C for 48 h. Positive colonies were observed to turn red to black.

The selected strains for EPS production were inoculated into MB medium containing 1 mM copper (250 mg/L) and incubated at 28°C for 72 h in a shaker at 150 rpm. The MB medium without copper served as a control. The cells were centrifuged at 8000 rpm for 10 min, and the supernatant was mixed with two volumes of cold 95% ethanol, followed by overnight incubation at 4°C. Most EPS was precipitated by centrifugation at 8000 rpm for 20 min at 4°C. It was then washed twice with cold 95% ethanol for purification dissolved in 10 mL of ddH_2_O and filtered through a 0.22-μm sterile filter (Whatman Uniflo, United States). The polysaccharide in EPS extract was determined using the phenol–sulfuric acid method with glucose as the standard (Dubois et al., 1951). The proteins in EPS extract were detected using the BCA Protein Assay Kit (Thermo Scientific, USA) with bovine serum albumin as the standard (Smith et al., 1985). All the optical density measurements were detected using a multimode microplate reader (Thermo Scientific, Model: Varioskan LUX, United States).

### 2.5 Determination of the cu (II) resistance ability and biosorption capacity of isolates

The resistance of the isolates to Cu was tested by determining their growth on solid and liquid MB medium with various concentrations of CuSO4 (0, 0.4, 0.8, 1.2, 1.6, 2, 4, 6, 7, 8, 9, 10 mM) at 28°C for 5 days in triplicate. Use the formula (R_i_ = ∣(V_0_-V_i_)/V_0_∣) to determine the effect of copper concentration on growth rates. In this formula, R_i_ represents the relative change in growth rate at an i mM copper concentration. V denotes the growth rate of the strains during the exponential growth phase, which is observed from 8 to 16 h in this study. V_0_ is the exponential growth rate at 0 mM copper concentration, serving as a baseline control. V_i_ is the exponential growth rate at the i mM copper concentration. Use statistical software SPSS to perform an analysis of variance (ANOVA) to assess the statistical significance of the growth rate differences under different copper concentrations among strains.

The tolerance of the bacteria against various concentrations of heavy metals was tested. To evaluate the sorption capability, isolates were cultured in triplicate in 100 mL of MB medium containing 1 mM Cu (250 mg/L) in a 250 mL flask at 150 rpm for 24 h at 28°C. The supernatants of cultures were sampled every 24 h, filtered, and then diluted with 10% HNO_3_ for the estimation of copper in the medium. To estimate the biosorption capacity of EPS, the extracted EPS was dissolved to a final concentration of 1 g/L, followed by the addition of different concentrations of CuSO_4_ solution ranging from 0 to 400 mg/L and after 1-h incubation then filter diluted with 10% HNO_3._ Copper ion concentrations were estimated using an atomic adsorption spectrometer (Srivastava and Majumder, 2008) and measured using an inductively coupled plasma optical emission spectrometer (ICP-OES, PerkinElmer, Model: Optima 7,300 V, United States). Using an uninoculated medium without CuSO_4_ standard solution served as a control.

### 2.6 Characterization of *Marinobacter metalliresistant* CuT 6

#### 2.6.1 Physiological characterization of *Marinobacter metalliresistant* CuT 6

The determination of the temperature range for growth was tested over the range of 4–45°C (i.e., 4, 10, 15, 20, 28, 32, 37, 40, 45, 50, and 55). The pH range for growth was tested from initial pH 4.0 to initial pH 10.0, at 28°C, with MES buffer (pH 4.0–6.0), PIPES buffer (pH 7.0–8.0), HEPES buffer (pH 8.0–9.0), and AMPSO buffer (pH 9.0–10.0). Salt tolerance was tested at 28°C in the MB medium prepared with various concentrations of NaCl (0–20% w/v, 1% intervals), which substitute for sea salts. The carbon substrate utilization patterns of the isolate were investigated using the BIOLOG Gen III microPlate in triplicates under optimal growth conditions. The enzymatic activities were assessed using API ZYM strips (bioMérieux). Membrane polar lipids for the PLFA analysis were extracted from freeze-dried biomass using acidic methanol, and their fatty acid composition was examined using GC–MS as previously described by Zhilina et al. (1997). The total phospholipids were identified by two-dimensional TLC as described by Sorokin et al. (2012). Respiratory lipoquinones were analyzed in cold acetone extract first by TLC (Collins, 1985) and then a major UV-absorbing band was eluted and subjected to tandem mass spectrometry (Thermo Fisher Scientific, Model: LCG Advantage Max, United States) with chemical ionization at atmospheric pressure. The quinones were identified by ionic mass by HPLC-MS. Morphological characteristics of cells of the novel isolate were determined by using light microscopy (Olympus, Model: CX21, Japan) and transmission electron microscopy (JEOL, Model: JEM-1230 and JEM2100, Japan).

#### 2.6.2 Genome analysis of *Marinobacter metalliresistant* CuT 6

The whole-genome sequencing was conducted by Gene Denovo Biotechnology Co., Ltd. (Guangzhou, China) using Illumina NovaSeq 6,000 (Illumina, San Diego, CA, USA) and PacBio Sequel II system (PacBio, Menlo Park, CA, United States) according to the manufacturer’s suggested protocols. Fastp version 0.20.0 and Pilon version 1.23 were used to control and correct the data (Walker et al., 2014; Chen et al., 2018). The complete genome was assembled using Falcon version 0.3.0(Chin et al., 2016). The obtained genome sequences were annotated using the NCBI Prokaryotic Genome Annotation Pipeline and deposited at DDBJ/ENA/GenBank. The tRNA genes were predicted using tRNAscan-SE version 1.3.1 (Lowe and Chan, 2016), and the rRNA genes using RNAmmer version 1.2 (Lagesen et al., 2007). The content of the genomic G + C was determined by the complete genome sequence.

#### 2.6.3 Phylogenetic analyses of *Marinobacter metalliresistant* CuT 6

The average nucleotide identity (ANI) of the genome was calculated with the algorithm of OrthoANIu using the EzBioCloud web service^3^ (Yoon et al., 2017b). The digital DNA–DNA hybridization (diDDH) estimate values were analyzed using the Genome-to-Genome Distance Calculator^4^ (Meier-Kolthoff et al., 2022). UBCG 3.0 analysis (Kim et al., 2021) based on a set of 92 single-copy core genes was performed to investigate the relationships among strain CuT 6 and the related species of *Marinobacte*r (*n* = 22). The UBCG utilizes a collection of external tools to derive a phylogenomic tree from a range of genomic sequences. These tools include Prodigal (Hyatt et al., 2010), Hmmsearch,^5^ Mafft (Katoh and Standley, 2013), RaxML (Stamatakis, 2014), and FastTree (Price et al., 2010).

### 2.7 Data availability

The GenBank/EMBL/DDBJ accession numbers for the 16S rRNA gene sequence of isolates are shown in Table 1. The whole-genome project for *M. metalliresistant* CuT 6 has been deposited in GenBank under the accession number ASM3809864v1 and is submitted in the NCBI under BioSample number SAMN29498721.

## 3 Results and discussions

### 3.1 Isolation and identification of copper-resistant bacteria

**Figure 1:**
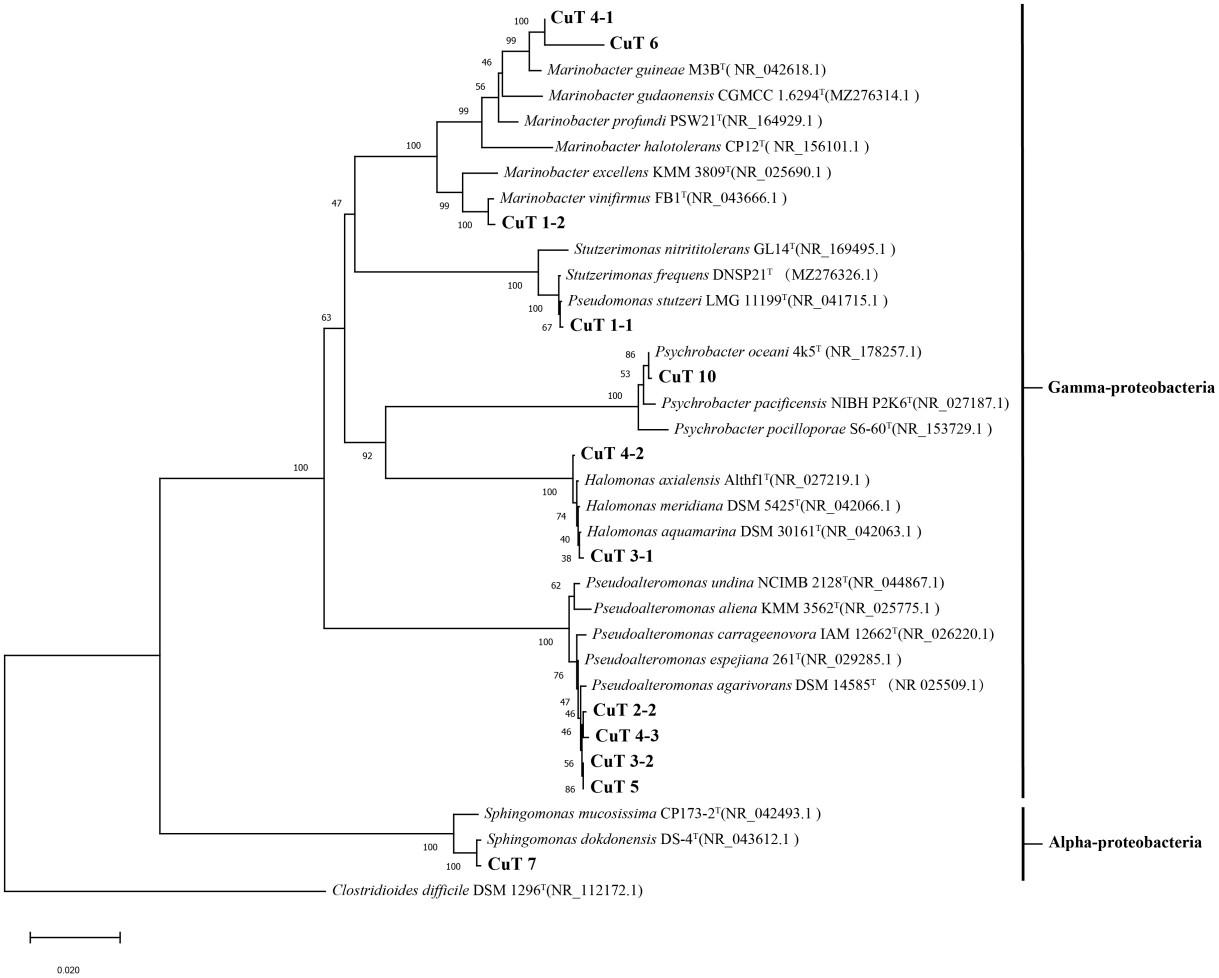
Phylogenetic tree reconstructed based on the small-subunit (16S) rRNA gene (1,399 bp) of isolates by the neighbor-joining method showed their positions and bootstrap values (out of 1,000 replicates).

Twelve copper-resistant bacteria were isolated from hydrothermal fields, of which 11 were assigned to the γ-Proteobacteria and one to the α-Proteobacteria as determined by their 16S rDNA sequences. They were affiliated with the *Pseudoalteromonas* (4), *Marinobacter* (3), *Halomonas* (2), *Psychrobacter* (1), *Pseudomonas* (1) genus in the γ-Proteobacteria group, and the *Sphingomonas* (1) genera in the α-Proteobacteria group (Figure 1; Table 1). All the isolates had growth temperatures between 4 and 37°C, with the optimal growth temperature being higher than 15°C, specifically approximately 28°C. As all the strains had growth at 4°C, they could be assigned as psychrotrophs. All 12 strains were Gram-negative, neutrophilic, and aerobic.

Among these genera, *Pseudomonas* and *Halomonas* strains have often been found heavy metal resistant ability and used to sequester heavy metals such as Cd (II), Cu (II), Cr (VI), and Ni (II) from aqueous solution (Gabr et al., 2008; Fang et al., 2011; Ozturk et al., 2012; Govarthanan et al., 2015; Mădălina et al., 2016; Chug et al., 2021). Strain CuT 1–1 closest to *Pseudomonas stutzeri* ATCC 17588 was isolated from sediments and tolerated with 9 mM copper. Two *Halomonas* strains CuT 3–1 and CuT 4–2 showed extra high copper-resistant capacity of up to 10 mM, which are close to extremely halotolerant bacteria *Halomonas meridiana* DSM 5425 isolated from Antarctic saline lake (Kaye and Baross, 2000; Jonathan et al., 2011).

*Pseudoalteromonas*, *Marinobacter,* and *Psychrobacter* are broadly distributed in marine environments, including hydrothermal vents, subseafloor, and deep-sea sediments (Holmstrom and Kjelleberg, 1999). However, little reported their metal-resistant ability. For example, the EPS-producing *Pseudoalteromonas* sp. MER144 from Antarctic seawater showed metal adsorption capacity (Caruso et al., 2017). In this study, three strains showed 99.57–99.93% similarity with marine *Pseudoalteromonas agarivorans* DSM 14585 and had high copper-resistant ability 9–10 mM. Another *Pseudoalteromonas* sp. strain CuT 3–2 showed 99.01% 16 s rDNA similarity with *Pseudoalteromonas carrageenovora* IAM12662. Three *Marinobacter* strains CuT 1–2, CuT 4–1, and CuT 6 were close to *M. vinifirmus* FB1^T^ (99.93% 16 s rDNA similarity) and *M. guineae* M3B^T^ (99.21, 96.88% 16 s rDNA similarity). Strain CuT 6 was identified as a novel species and further characterized as follows. Strain CuT 10 had 98.89% 16 s rDNA similarity with *Psychrobacter oceani* 4k5^T^, which was isolated from marine sediment.

*Sphingomonas* genus in the α-Proteobacteria group was frequently isolated from soil and encoded heavy metal resistance-related genes such as multicopper oxidase and the czc efflux system (Altimira et al., 2012; Kim et al., 2020). Those strains possess multifaceted functions ranging from remediation of environmental contaminations to promoting plant growth (Asaf et al., 2020). In this study, strain CuT 7 closest to *Sphingomonas dokdonensis* DS-4 was isolated. *Sphingomonas dokdonensis* DS-4 was isolated from soil, but no metal-resistant ability was found previously (Yoon et al., 2006).

Diverse and adaptable heterotrophic α- and γ-proteobacteria have consistently been detected and repeatedly isolated from different venting areas throughout a wide range of environmental conditions in hydrothermal plumes, sulfide structures, and associated sediments, demonstrating their prevalence and dominance in the deep-sea hydrothermal field (Edwards et al., 2003; Kato et al., 2013; Meier et al., 2016; He et al., 2022). In the sample area (Southwest Indian Ocean Ridge), metagenomic analysis also showed that α- and γ-proteobacteria were abundant in two sulfides samples, accounting for 7.79–24.54% and 20.6–61.0% (Zhong et al., 2022). Our findings implied that their adaptability might encompass their remarkable resistance ability to heavy metals.

### 3.2 Copper resistance profile and adsorption capacity by isolates

In this study, all 12 isolates showed highly copper-resistant activities up to 6 to 10 mM in the liquid MB medium with various concentrations of CuSO_4_ (Table 1; Supplementary Figure S1). Four strains, including *Halomonas* sp. CuT 3–1 and CuT 4–2, *Pseudoalteromonas* sp. CuT 4–3 and CuT 5, could tolerate high concentrations of Cu (II) up to 10 mM. Among the three isolated *Marinobacter* strains (CuT 1–2, CuT 4–1, and CuT 6), strain CuT 6 exhibited an exceptional capacity to withstand copper concentrations reaching as high as 8 mM. These three distinct genera, including *Halomonas* sp. CuT 3–1, *Pseudoalteromonas* sp. CuT 4–3, and *Marinobacter metalliresistant* CuT 6, were chosen as representatives for the analysis of their copper-resistant abilities, as they exhibited high levels of copper resistance.

**Figure 2:**
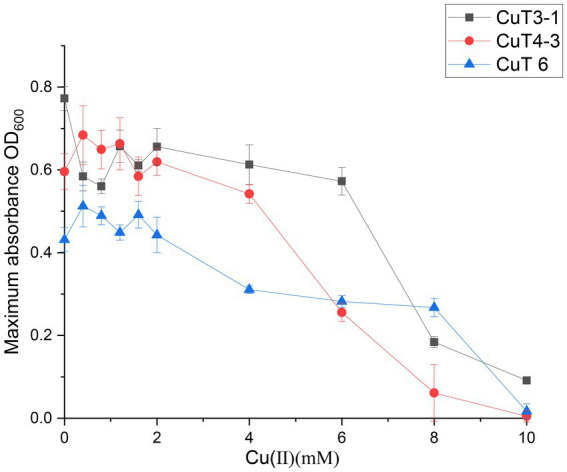
Highest growth number of highly copper-resistant strains, including *Halomonas* sp. CuT 3–1, *Pseudoalteromonas* sp. CuT 4–3 and *Marinobacter metalliresistant* CuT 6, were grown in the MB medium supplemented with the concentration of CuSO_4_ at 28°C for 85 h.

**Figure 3:**
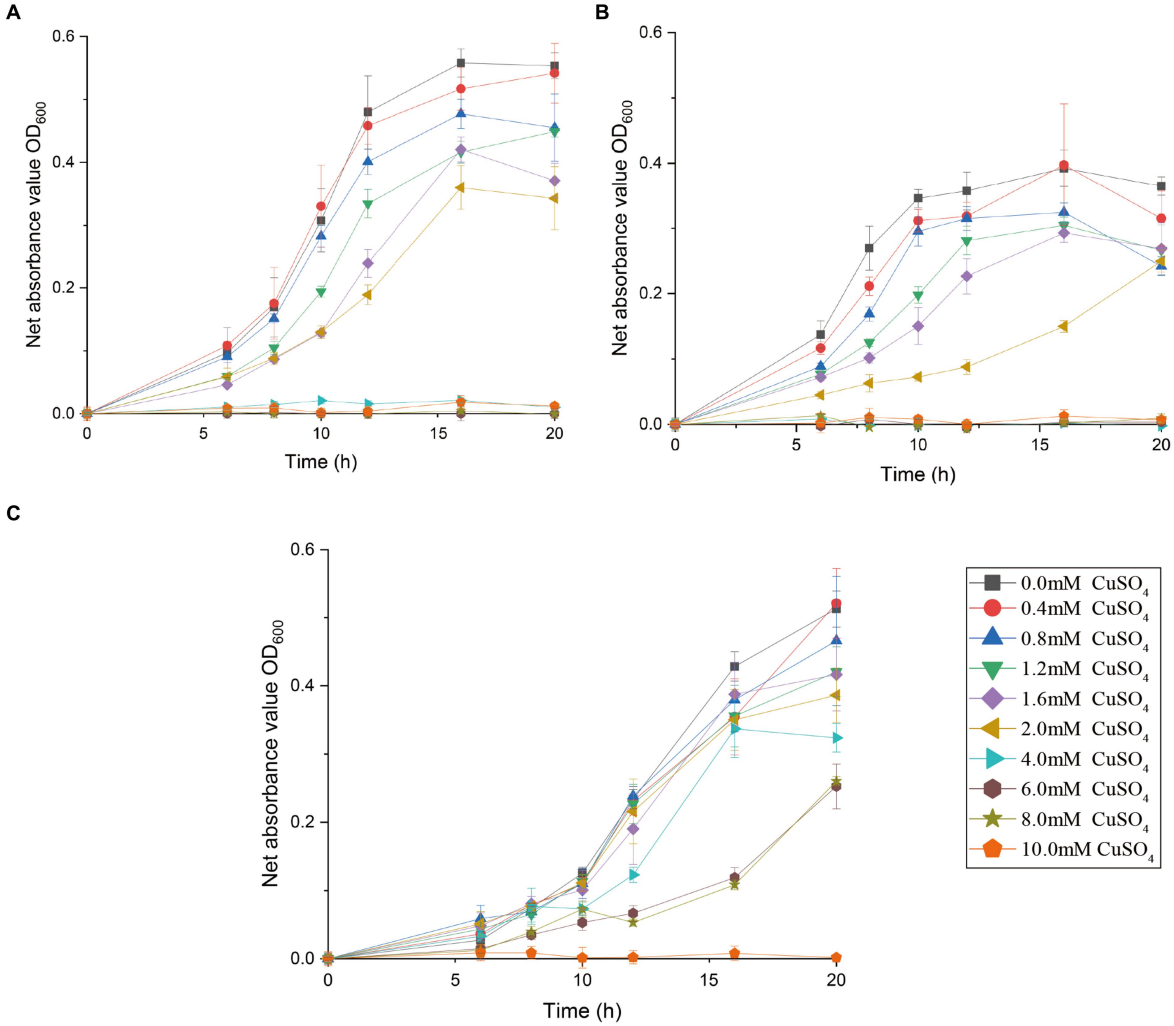
Growth curve of bacterium *Marinobacter metalliresistant* CuT 6 **(C)**, in comparison to its phylogenetically closest relatives, *M. guineae* M3B^T^ **(A)** and *M. profundi* PSW21^T^ **(B)**. Strains were grown at 28°C for 20 h in MB medium supplemented with 0–10 mM CuSO_4_. Growth was observed using turbidity (OD_600_).

The effects of copper with treatment concentrations ranging from 0 to 10 mM on the bacterial growth curve and the maximum bacterial growth number are shown in Supplementary Figure S1 and Figure 2. The growth of *Halomonas* sp. CuT 3–1 was inhibited in the presence of higher than 2 mM copper (Supplementary Figure S1A). Furthermore, it could have growth with OD600 (optical density at 600 nm) of 0.09 in the 18 h culture with 10 mM Cu (II) (Supplementary Figure S1A). There was almost no effect on the highest cell growth number of *Pseudoalteromonas* sp. CuT 4–3 under 2.0 mM Cu (II) and *M. metalliresistant* CuT 6 under 1.6 mM Cu (II) (Figure 2). *Pseudoalteromonas* sp. CuT 4–3 could grow up to OD600 = 0.3 at 6 mM Cu (II) (Supplementary Figure S1B). *M. metalliresistant* CuT 6 showed the highest growth in MB with 0.4–0.8 mM Cu (II) and reached an optical density (OD600) of 0.25 with 8 mM Cu (II). Previous reports showed that most copper-resistant bacteria tolerate 0.5–3.5 mM of Cu (II) (Altimira et al., 2012). *Bacillus* sp. showed the highest copper-resistant ability up to 5.5 mM (Kunito et al., 1997). Some copper-resistant bacteria could decrease copper (<1.5 mM) for the remediation of metal-contaminated waters or soils (Kugler et al., 2022). The isolates in this study displayed high resistance to Cu (II) up to 6–10 mM, which indicated their highly heavy metal resistant ability might contribute to their adaptation in the hydrothermal fields. Besides, the genus *Marinobacter* is very broadly distributed in marine and terrestrial environments (Cooper et al., 2022). However, only one report demonstrated that *Marinobacter adhaerens* HP15 had zinc resistance by two CzcCBA efflux pumps and helped it to colonize aggregates (Stahl et al., 2015; Will et al., 2020). In this study, typical heavy metals, including Cu (II), Cr (VI), Cd (II), Co (II), Zn (II), and Hg (II), were further determined to study the heavy metal-resistant abilities of 30 *Marinobacter* species (28 type species and 2 new isolates from hydrothermal vents) (Supplementary Figure S2). Most of them exhibited certain copper resistance, with a minimum tolerance of 2 mM and a maximum tolerance of 6 mM Cu (II). Among them, strain CuT 6 showed the highest copper resistance capacity up to 8 mM and could tolerate 2 mM Co (II), 3 mM Zn (II), and 0.3 mM Hg (II) (Figure 3; Supplementary Figure S2). Among their phylogenetically closest relatives, *M. guineae* strain M3B^T^ and *M. profundi* strain PSW21^T^ demonstrated the highest copper resistance at a concentration of only 2 mM, which is different from the growth rate changes observed in CuT 6 when CuT 6 was exposed to copper. The differences between strains were especially obvious at concentrations of 4, 6, and 8 mM. (*p* < 0.05, Table. S6). Besides, each of *Marinobacter* spp. displayed resistance to at least one type of heavy metal. Among them, *Marinobacter fuscus* NH169-3^T^ showed metal-resistant ability to all the tested heavy metals.

### 3.3 Biosorption of copper by produced EPS of isolates

Except for strain CuT 3–2, 11 out of 12 isolates exhibited positive EPS production ability, forming red or black colonies on the CRA plates compared with colonies on MB plates (Supplementary Figure S3). The EPS was subsequently purified from a 24-h liquid culture of *Halomonas* sp. CuT 3–1, *Pseudoalteromonas* sp. CuT 4–3, and *M. metalliresistant* CuT 6 as described methods (Maalej et al., 2015).

Compared to the absence of Cu (II) in the medium, the addition of 1 mM Cu (II) significantly induced the production of EPS by these three isolates, which were mainly composed of proteins and sugars (Supplementary Figure S4). The increase in the concentration of EPS from 379.65 mg/L to 1013.00 mg/L in the culture of *Halomonas* sp. strain CuT 3–1 with copper stimulation. The polysaccharide and the protein contents in the EPS of CuT 3–1 increased by 3.64-fold [from 21.64 mg/L (5.70%) to 100.49 mg/L (9.92%)] and 2.55-fold [from 358.04 mg/L (94.30%) to 912.06 mg/L (90.08%)] with copper stimulation. Contents of EPS of *Pseudoalteromonas* sp. strain CuT 4–3 consistently increased from 366.70 mg/L to 1057.92 mg/L as Cu increased. The polysaccharide content of strain CuT 4–3 increased from 33.37 mg/L (9.10%) to 66.12 mg/L (6.25%), and the protein part increased from 333.44 mg/L (90.10%) to 992.00 mg/L (93.75%). The total EPS content of strain CuT 6 was lower than those of strain CuT 3–1 and strain CuT 4–3. The EPS content of strain CuT 6 increased a little from 214.35 mg/L to 447.17 mg/L with copper stimulation. The copper ion addition also induced an increase in polysaccharide content [from 50.48 mg/L (23.55%) to 82.19 mg/L (18.38%)] and protein content [from 214.31 mg/L (76.45%) to 364.96 mg/L (81.62%)] of strain CuT 6. In addition, we examined the composition and contents of EPS produced by strain CuT 6 and its closely related type strains under conditions with and without copper. The results indicated that strain CuT 6 produced different components and contents of EPS, and it responded more sensitively to copper stimulation compared to the other five *Marinobacter* strains (Supplementary Figure S10).

**Figure 4:**
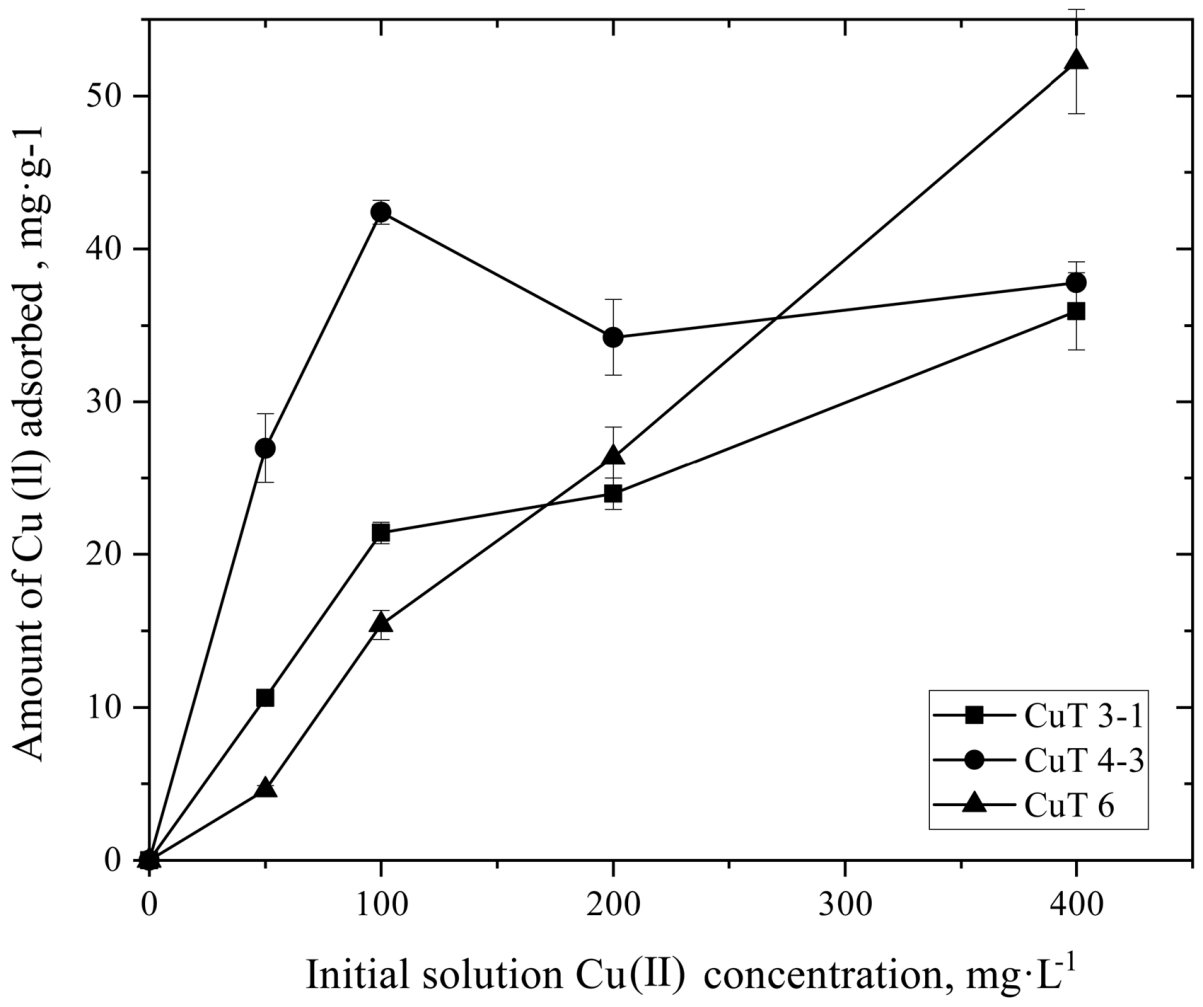
Biosorption of copper by the crude exopolysaccharide (EPS) produced by *Halomonas* sp. CuT 3–1, *Pseudoalteromonas* sp. CuT 4–3 and *Marinobacter metalliresistant* CuT 6 over a range of metal concentration (0–400 mg/L of Cu (II)).

The copper adsorption capacity of the culture medium of copper-tolerant bacteria during growth at 250 mg/L of CuSO_4_ was monitored (Supplementary Figure S5). The binding ability of EPS produced by these bacteria at different concentrations of 0–400 mg/L of CuSO_4_ was also determined, as shown in Figure 4. With the addition of CuSO_4_, the copper ion content in the mediums was reduced by copper-resistant bacterial strains. After 24 h, strain CuT 3–1 reduced 75.38 mg/L of CuSO_4_. After 48-h cultivation, strain CuT 4–3 and strain CuT 6 reduced 74.60 mg/L and 79.21 mg/L of CuSO_4_, respectively. The binding of metal by EPS increased linearly with the content of Cu (II). The EPS of *M. metalliresistant* CuT 6 was found to have the highest binding ability up to 52.24 mg CuSO_4_/g EPS. The maximum amount of Cu (II) bound to the EPS of strain CuT 4–3 and CuT 3–1 was 37.78 and 35.92 mg CuSO_4_/g EPS. These three copper-tolerant bacterial strains all exhibited abilities to reduce CuSO_4_ concentrations in the medium. However, their EPS adsorption capacities varied significantly. The EPS produced by strain CuT 6 had a higher capacity to adsorb CuSO_4_ than those produced by the other two strains. The results indicated that the copper ions could be adsorbed by the cells themselves and by the secreted EPS. The findings revealed that upon exposure to copper, the production of EPSs by copper-resistant bacteria increased, along with a fluctuating ratio of polysaccharides and proteins within the EPS matrix. Notably, the EPS surrounding the cells displayed remarkable adsorption capabilities for heavy metals, which might contribute to bacterial adaptation in the harsh environments of deep-sea hydrothermal vents.

### 3.4 Characterization of *Marinobacter metalliresistant* CuT 6

#### 3.4.1 Phylogenetic and genomic analysis of *Marinobacter metalliresistant* CuT 6

The maximum-likelihood phylogenetic tree based on the 16S rRNA gene and the genome-based phylogenomic tree by using the up-to-date bacterial core gene sets (92 genes) were established to elucidate the phylogenetic position of strain CuT 6 in the phylum of Proteobacteria. It showed that strain CuT 6 belonged to the genus *Marinobacter* and was closest to *M. guineae* and *M. profundi* (Figure 1; Supplementary Figure S6). Phylogenetic analysis of the 16S rRNA gene sequences showed that strain CuT 6’s closest relative was *M. guineae* M3B^T^, with which it shared 99.1% similarity. The ANI values of strain CuT 6 to *M. guineae* M3B^T^, *M. profundi* PSW21^T,^ and *M. lipolyticus* SM19^T^ were 88.3, 76.91, 77.3%, respectively, which are below the standard ANI criterion for species identity (95.0–96.0%) (Kim et al., 2014). The diDDH estimate values between strain CuT 6 and *M. guineae* M3B^T^ (Montes et al., 2008), *M. lipolyticus* SM19^T,^ and *M. gudaonensis* CGMCC 1.6294^T^ were 43.2 ± 6.5%, 21.7 ± 0.9%, and 30.2 ± 6.6% (Table S5), respectively. These values were far below the standard criterion (70%) for the delineation of prokaryotic species (Stackebrandt and Goebel, 1994). These results confirm that strain CuT 6 represents a novel species of the genus *Marinobacter*.

The genome of strain CuT 6 consists of a single circular 4,411,896 bp chromosome without an extrachromosomal element. A total of 1,949 genes and 1,897 protein-coding sequences were predicted. Nine rRNA loci and 50 tRNA genes were identified. The G + C content of the genomic DNA of strain CuT 6 was 57.60%, calculated *in silico* using the complete genome sequence of strain CuT 6. The genome characteristics of CuT 6 and its relatives are listed in Table S4.

#### 3.4.2 Physiological characterization of *Marinobacter metalliresistant* CuT 6

Cells of strain CuT 6 are rod-shaped, measuring 0.6 μm wide and 2.0–3.0 μm long. They are Gram-negative and non-spore-forming (Supplementary Figure S7). Cells are motile with the polar flagella. Growth was observed between 4°C and 45°C, and the optimal temperature for growth was 28°C. Growth was observed from 0 to 15% (w/v) NaCl. The optimal salt concentration was 3–4% for NaCl. The novel isolate is obligately chemoorganoheterotrophic, and its growth is observed under aerobic or anaerobic conditions. Strain CuT 6 was found to be able to utilize carbohydrates (including dextrin gentiobiose L-fucose, D-fucose, D-mannitol, glycerol, and D-galacturonic acid), organic acids (L-lactic acid, α-ketoglutaric acid L-malic acid, D-malic acid, β-hydroxybutyric acid, acetoacetic acid, propanoic acid, and trisodium citrate), and amino acids (including L-alanine, L-glutamic acid, and L-Histidine), but not L-arabinose, D-mannose, maltose, potassium gluconate, adipic acid, or capric acid. Nitrate and nitrite are reduced. Tween 40 is hydrolysed, but lecithin, casein and starch are not. Using the API 20NE test, it was found that D-glucose fermentation is positive, but gelatin hydrolysis, indole production, glucose, arginine dihydrolase, and urease are negative, which was different from *M. guineae* M3B^T^. Strain CuT 6 could produce alkaline phosphatase, esterase (C4), esterase lipase (C8), lipase (C14) leucine arylamidase, valine arylamidase, cystine arylamidase, acid phosphatase, naphthol-AS-BI-phosphohydrolase and N-acetyl-b-glucosaminidase, but not α-galactosidase, β-galactosidase, β-glucuronidase, α-glucosidase, β-glucosidase, α-mannosidase, and α-fucosidase (API ZYM). The main fatty acids are summed feature 3 (C_16:1_ ω7c and/or iso-C_16:1_ ω6c), C_16:1_ ω9c, C_16:0_, and C_18:1_ ω9c. The major respiratory quinone of strain CuT 6 was identified as ubiquinone 9 (Q-9 and Q-9(H2)). The polar lipids of strain CuT 6 were identified as phosphatidylethanolamine (PE), phosphatidylglycerol (PG), diphosphatidylglycerol (GL), and aminolipid (AL), as shown in Supplementary Figure S8. Differences in physiological, biochemical, and chemotaxonomic characteristics between strain CuT6 and its relatives are given in Table 2.

**Table 2:** Comparison of the phenotypic characteristics of strain CuT 6 and their closely related reference strains.

Characteristic	1	2	3
Growth at/in			
Minimum temperature (°C)	4	4	4
Maximum temperature (°C)	45	42	45
NaCl range (%)	1–15	1–15	0.5–20
DNA G + C content (mol%)	57.6%	57.1%	60.3%
Hydrolysis of:			
Starch	−	−	−
Urea	−	−	−
Nitrate reduction to nitrite	+	+	+
Nitrite reduction to N2	+	+	+
Enzyme activities*:			
Esterase C4	+	+	+
Esterase lipase C8	+	+	+
Esterase lipase C14	+	+	+
Valine arylamidase	+	+	+
Cystine arylamidase	+	-†	+
Acid phosphatase	+	+	W‡
Naphthol-AS-BI-phosphohydrolase	+	+	-‡
N-acetyl-β-glucosaminidase	−	−	+‡
Trypsin	−	−	−
α-Chymotrypsin	−	−	−
Utilization of*:			
Glycerol	+	+	−
D-Glucose	+	+	−
D-Fructose	W	+	−
D-Mannitol	+	−	+
Maltose	−	−	−
Citric acid	−	−	−
D-Gluconic acid	−	+	−
Gentiobiose	+	−	−
L-Alanine	+	−	−
L-Glutamic acid	+	−	+
L-Histidine	+	−	−
α-Keto-Glutaric acid	+	−	+

*Data from this study. †Data from this study are different from Montes et al. (2008). ‡Data from this study are different from Cao et al. (2019). Negative; +, positive; W, weakly positive; ND, no data available. Taxa: 1. *M. metalliresistant* CuT 6^T^ 2. *M. guineae* M3B^T^ 3. *M. profundi* PSW21^T^.

#### 3.4.3 Possible mechanisms of copper resistance in *Marinobacter metalliresistant* CuT 6

Microbial copper resistance systems span copper efflux (*cop, pco, cus, cue*, and other extrachromosomal efflux systems), copper sequestration (*cusF* and siderophores), and copper oxidation (mixed copper oxidases and superoxide dismutase) (Rademacher and Masepohl, 2012). *CopA* is considered to be a marker gene for copper-tolerant microorganisms, which mainly mediates copper ion efflux reactions in microorganisms. *CopA* is divided into two groups encoding for multicopper oxidase and P-type ATPase (Li et al., 2015). In addition, the *Mer* family genes are not only involved in enhancing microbial mercury tolerance but are also thought to act as regulatory factors for genes conferring copper resistance, such as *MerR* (Li et al., 2014). Other multiple heavy metal resistance genes include the cobalt–zinc–cadmium efflux system (*czcABCD*), arsenic (*arsABCR*), and cation efflux genetic system (*cusAB*) (Silver and Phung, 1996).

**Figure 5:**
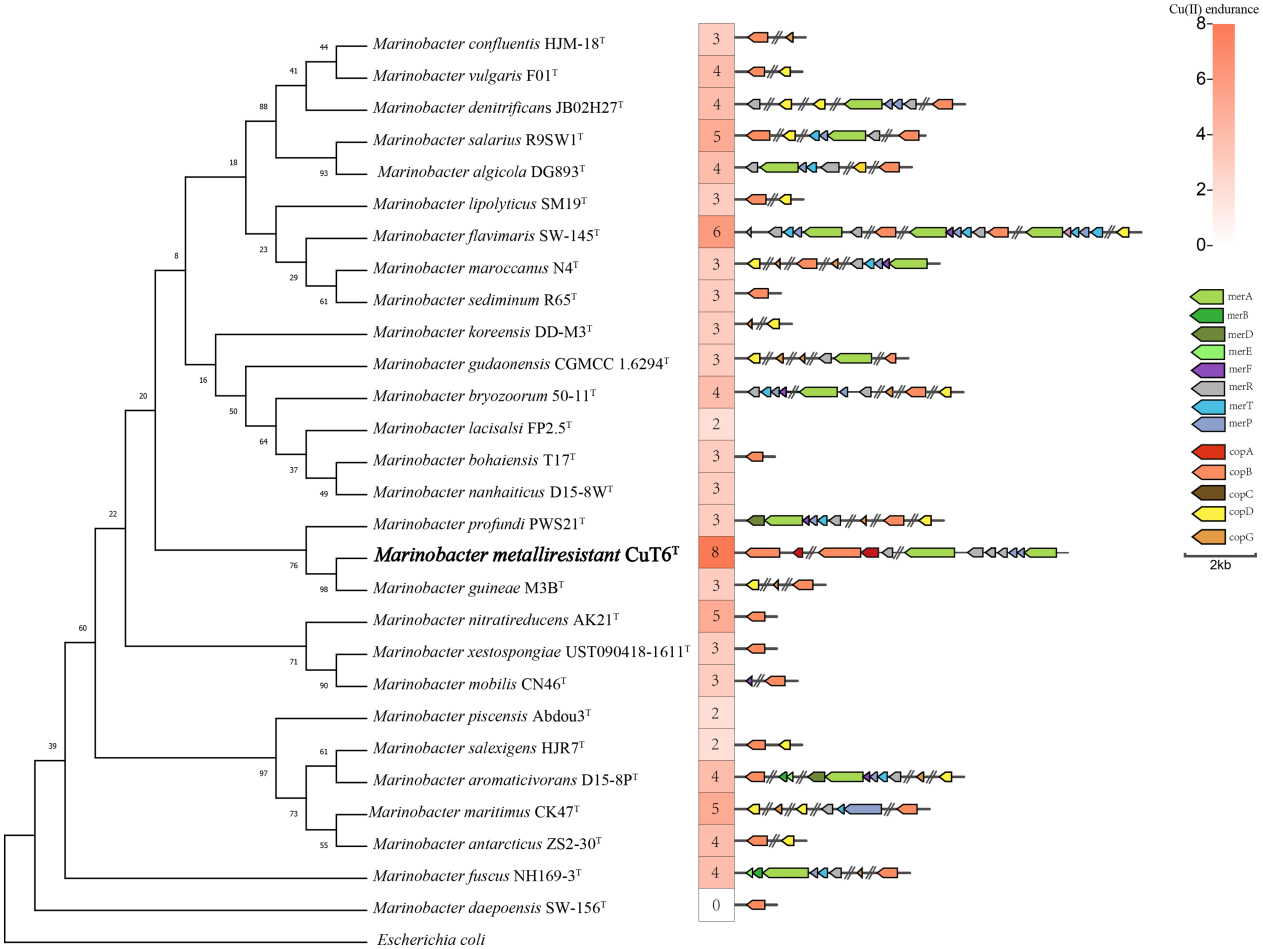
Maximum-likelihood phylogenetic tree reconstructed based on the 16S rRNA gene sequences of strain CuT 6 and representative members in the genus *Marinobacter*, with their copper resistant ability and related genes. *Escherichia coli* (NR_024570.1) was selected as an outgroup. Bootstrap values are given on the nodes of the tree. Branch node values below 50 are not shown here. There were a total of 1,249 positions in the final dataset. Bar, 0.01 substitutions per nucleotide position.

We searched the copper resistance-related genes in the genus *Marinobacter* genomes including strain CuT 6 and other type strains (Figure 5; Table S2). Based on an analysis of the genomic copper resistance genes, it was discovered that strain CuT 6 uniquely encoded four copies of the *copA* gene, encompassing two multicopper oxidases and two ATPases, as well as a single *copB* gene within its genome (Supplementary Figure S9). It also encoded the copper resistance protein PcoA (NLK58_09295), which is required for the copper-inducible expression of copper resistance. This is consistent with its high copper-resistant ability. In addition, at least one Mer operon (NLK58_12425-NLK58_12440), including mercuric reductase (*merA*), was harbored by strain CuT 6. Additionally, strain CuT6 contains a partial cation efflux genetic system (*cusAB*) and cobalt–zinc–cadmium efflux system (*czcAR*), such as two *cusA* (NLK58_09210 and NLK58_12420) and two *czcA* (NLK58_09270 and NLK58_16520) genes (Table S2). These efflux genes within strain CuT 6, including *copA* (ATPases)*, copB, cusAB*, *czcA*, and *MerPT,* potentially play a key role in the heavy metal transport capabilities. The enzymes *copA* (multicopper oxidase) and *pcoA* are responsible for oxidizing copper. In the *M. metalliresistant* CuT 6 genome, we have also identified some potential EPS synthesis-related genes and gene clusters, mainly composed of glycosyltransferases, forming functional structures for polysaccharide synthesis (Table S3).

As reported in the genome of *Pseudoalteromonas* sp. CuT 4–3 by Ji et al. (2024), our study by genomic analysis concluded that multiple heavy metal resistance genes as active transport or efflux mechanisms and EPS biosynthesis for the absorption of these copper-resistant bacteria were responsible for heavy metal detoxification. These genes included copper-resistant genes (*copABCD* and *pcoAB*), cobalt–zinc–cadmium efflux system protein (*czcABCD*), arsenic (*arsABCR*), and cation efflux genetic system (*cusAB*), as well as proteins involved in polysaccharide syntheses such as the epsG family, cellulose biosynthesis bcs operons, Wzx flippase, Wzy polymerase, and polysaccharide copolymerase (Wzz) proteins.

#### 3.4.4 Description of *Marinobacter metalliresistant* sp.nov

The cells are motile, round-ended rods (2.0–3.0 μm in length, 0.6 μm in width) with flagellum. Endospores are never observed. Growth is observed at salinities from 0 to 15% (optimum 3–4%), from pH 5 to 8 (optimum 7), and at temperatures between 4 and 45°C (optimum 28°C). Doubling time is 56 min under optimal growth conditions. Aerobic or facultative anaerobe. Uses complex organic compounds and organic acids including yeast extract, peptone, tryptone, casein, casamino acids, citrate, lactate, acetate, fumarate, propanoate and pyruvate. Does not reduce sulfite, sulfate, thiosulfate or nitrate. The type strain, CuT 6^T^ (=MCCC M25622^T^ = KCTC 92387^T^), was isolated from a hydrothermal sulfide sample collected at a depth of 2,901 m in a hydrothermal area of the southwest Indian Ocean (50.75 E, 40.04 S). The DNA G + C content of the type strain is 57.6 mol%.

## 4 Conclusion

Twelve highly copper-resistant bacteria were isolated from hydrothermal fields, which tolerate copper concentrations of up to 6–10 mM. They were affiliated with α-Proteobacteria and γ-Proteobacteria, including *Sphingomonas* (1), *Pseudoalteromonas* (4), *Marinobacter* (3), *Halomonas* (2), *Psychrobacter* (1), and *Pseudomonas* (1). The EPS content of strains was induced by copper ion, which could absorb 40 to 50 mg•g^−1^ copper. By physiologic and genomic analysis, we concluded that the copper resistance mechanisms, mainly the production of exopolymeric substances, and copper resistance genes as active transport or efflux mechanisms, of these strains in deep-sea hydrothermal fields. A novel species, *Marinobacter metalliresistant* sp. nov. CuT 6^T^, was identified.

## Data Availability

The datasets presented in this study can be found in online repositories. The names of the repository/repositories and accession number(s) can be found in the article/Supplementary material.

## Footnotes

^1^https://mccc.org.cn/

^2^https://www.ezbiocloud.net/

^3^https://www.ezbiocloud.net/tools/ani, accessed on 1 May 2023.

^4^GGDC 3.0, https://ggdc.dsmz.de/, accessed on 7 May 2023.

^5^http://hmmer.org
