# Supplementary material for: Isolation of highly copper-resistant bacteria from deep-sea hydrothermal fields and description of a novel species Marinobacter metalliresistant sp. nov
Source: Front Microbiol. 2024 Aug 21;15:1390451. doi: 10.3389/fmicb.2024.1390451 (PMC11371751; doi:10.3389/fmicb.2024.1390451)

**Table S1**. Sample locations

| **Strains** | **Samples** | **Location** | **Depth(m)** | **Description** |
| --- | --- | --- | --- | --- |
| CuT 1-1 | DY65-SWIR-S046-TVG14 | E51°30’, S37°26’ | 2809 | Sediments |
| CuT 1-2 | DY65-SWIR-S046-TVG14 | E51°30’, S37°26’ | 2809 | Sediments |
| CuT 2-2 | DY65-SWIR-S021-TVG06 | E50°38’ ,S37°35’ | 1920 | Oxides |
| CuT 3-1 | DY65-SWIR-S048-TVG16 | E51°43’ ,S37°25’ | 2575 | Sediments |
| CuT 3-2 | DY65-SWIR-S048-TVG16 | E51°43’ ,S37°25’ | 2575 | Sediments |
| CuT 4-1 | DY65-SWIR-S013-TVG05 | E49°17’, S37°55’ | 2653 | Sulfides |
| CuT 4-2 | DY65-SWIR-S013-TVG05 | E49°17’ ,S37°55’ | 2653 | Sulfides |
| CuT 4-3 | DY65-SWIR-S013-TVG05 | E49°17’ ,S37°55’ | 2653 | Sulfides |
| CuT 5 | DY65-SWIR-S052-TVG19 | E51°42’ ,S37°26’ | 2019 | Sediments |
| CuT 6 | DY65-SWIR-S044-TVG12 | E50°38’ ,S37°35’ | 1875 | Oxides |
| CuT 7 | DY65-SWIR-S022-TVG07 | E49°15.’ ,S37°55’ | 1587 | Sediments |
| CuT 8 | DY65-SWIR-S036-TVG08 | E49°15’ ,S37°57’ | 1445 | Sulfides, Oxides |
| CuT 10 | DY65-SWIR-S060-TVG24 | E51°11’ ,S37°20’ | 1655 | Sediments |

**Table S2** Genes potentially involved in heavy metal resistance in the genome of *Marinobacter metalliresistant* CuT 6

| **Family** | **ID** | **Symbol** | **Nr-annotation** |
| --- | --- | --- | --- |
| *Cop* | NLK58_02490 | *copA* | copper resistance system multicopper oxidase |
|  | NLK58_09520 | *copA* | copper resistance system multicopper oxidase |
|  | NLK58_12365 | *copA* | copper-translocating P-type ATPase |
|  | NLK58_13925 | *copA* | copper-translocating P-type ATPase |
|  | NLK58_02485 | *copB* | copper resistance system membrane protein B |
|  | NLK58_09290 | *copB* | copper resistance system membrane protein B |
|  | NLK58_09525 | *copB* | copper resistance system membrane protein B |
|  | NLK58_00690 | *copG* | *CopG* family transcriptional regulator |
|  | NLK58_10835 | *copG* | *CopG* family transcriptional regulator |
|  | NLK58_14925 | *copG* | ribbon-helix-helix protein, *CopG* family |
|  | NLK58_19280 | *copG* | ribbon-helix-helix protein, *CopG* family |
| *pcoA* | NLK58_09295 | *pcoA* | copper resistance system multicopper oxidase |
| *Mer* | NLK58_12270 | *merA* | FAD-dependent oxidoreductase |
|  | NLK58_12440 | *merA* | mercury(II) reductase |
|  | NLK58_09560 | *merR1* | Cd (II)/Pb (II)-responsive transcriptional regulator |
|  | NLK58_12375 | *merR1* | Cd (II)/Pb (II)-responsive transcriptional regulator |
|  | NLK58_12395 | *merR* | Hg (II)-responsive transcriptional regulator |
|  | NLK58_12425 | *merR* | Hg (II)-responsive transcriptional regulator |
|  | NLK58_12435 | *merP* | mercury resistance system periplasmic binding protein *MerP* |
|  | NLK58_12430 | *merT* | mercuric ion transporter *MerT* |
| *cus* | NLK58_09210 | *cusA* | Putative silver efflux pump |
|  | NLK58_12420 | *cusA* | Putative silver efflux pump |
|  | NLK58_12415 | *cusB* | Membrane-fusion protein |
| *czc* | NLK58_09270 | *czcA* | Putative silver efflux pump |
|  | NLK58_16520 | *czcA* | Putative silver efflux pump |
|  | NLK58_14155 | *czcR* | response regulator |
| *pac* | NLK58_09345 | *pacS* | copper-translocating P-type ATPase |
|  | NLK58_09420 | *pacS* | copper-translocating P-type ATPase |
| *act* | NLK58_09330 | *actP* | copper-translocating P-type ATPase |
| *nos* | NLK58_08905 | *nosL* | copper-binding protein |
| Other | NLK58_09300 | *--* | copper-binding protein |

**Table S3** Genes potentially involved in EPS production in the genome of *Marinobacter metalliresistant* CuT 6.

| **Family** | **ID** | **Symbol** | **Nr-annotation** |
| --- | --- | --- | --- |
| *tol* | NLK58_16680 | *tolC* | Outer membrane protein |
|  | NLK58_03885 | *tolB* | Periplasmic component of the Tol biopolymer transport system |
|  | NLK58_03895 | *tolR* | Biopolymer transport protein |
| *arc* | NLK58_14700 | *arcA* | Arginine deiminase |
|  | NLK58_14710 | *arcC* | Carbamate kinase |
| *eps* | NLK58_16405 | *epsE* | Type II secretory pathway, ATPase PulE/Tfp pilus assembly pathway, ATPase PilB |
|  | NLK58_05210 | *epsE* | Type II secretory pathway, ATPase PulE/Tfp pilus assembly pathway, ATPase PilB |
| glycosyl  transferase | NLK58_03495 | *tuaG* | Glycosyltransferases involved in cell wall biogenesis |
|  | NLK58_01820 | *mgtA* | Glycosyltransferase |
|  | NLK58_02330 | *arnT* | 4-amino-4-deoxy-L-arabinose transferase and related glycosyltransferases of PMT family |
|  | NLK58_02335 | *sll0501* | Glycosyltransferases involved in cell wall biogenesis |
|  | NLK58_03455 | *alr2836* | Glycosyltransferases involved in cell wall biogenesis |
|  | NLK58_03460 | *RF_0543* | Glycosyltransferases involved in cell wall biogenesis |
|  | NLK58_03475 | *--* | -- |
|  | NLK58_03490 | *--* | Glycosyltransferase |
|  | NLK58_03495 | *tuaG* | Glycosyltransferases involved in cell wall biogenesis |
|  | NLK58_03555 | *pimC* | Glycosyltransferase |
|  | NLK58_03565 | *--* | -- |
|  | NLK58_03600 | *mshA* | Glycosyltransferase |
|  | NLK58_03605 | *--* | -- |
|  | NLK58_03610 | *--* | Glycosyltransferases, probably involved in cell wall biogenesis |
|  | NLK58_03625 | *--* | -- |
|  | NLK58_03640 | *MJ1059* | Glycosyltransferase |
|  | NLK58_03650 | *ydaM* | Glycosyltransferases, probably involved in cell wall biogenesis |
|  | NLK58_03680 | *wecA* | Sugar transferases involved in lipopolysaccharide synthesis |
|  | NLK58_06035 | *NGR_a03550* | Glycosyltransferases involved in cell wall biogenesis |
|  | NLK58_06100 | *--* | Glycosyltransferase |
|  | NLK58_06125 | *--* | -- |
|  | NLK58_06165 | *--* | Glycosyltransferase |
|  | NLK58_06215 | *pglA* | Glycosyltransferase |
|  | NLK58_08635 | *mgs* | Glycosyltransferase |
|  | NLK58_14610 | *arnT* | 4-amino-4-deoxy-L-arabinose transferase and related glycosyltransferases of PMT family |
|  | NLK58_14620 | *arnC* | Glycosyltransferases involved in cell wall biogenesis |
|  | NLK58_14635 | *arnT* | 4-amino-4-deoxy-L-arabinose transferase and related glycosyltransferases of PMT family |
|  | NLK58_14640 | *sll0501* | Glycosyltransferases involved in cell wall biogenesis |
|  | NLK58_16565 | *gspA* | Lipopolysaccharide biosynthesis proteins, LPS:glycosyltransferases |
|  | NLK58_16570 | *gspA* | Lipopolysaccharide biosynthesis proteins, LPS:glycosyltransferases |
|  | NLK58_16580 | *opsX* | ADP-heptose:LPS heptosyltransferase |
|  | NLK58_16595 | *wcaL* | Glycosyltransferase |
|  | NLK58_16635 | *--* | -- |
|  | NLK58_16650 | *RT0209* | Glycosyltransferases involved in cell wall biogenesis |
|  | NLK58_16655 | *mshA* | Glycosyltransferase |
|  | NLK58_16660 | *mshA* | Glycosyltransferase |
|  | NLK58_18270 | *mgtA* | Glycosyltransferase |
|  | NLK58_20280 | *arnC* | Glycosyltransferases involved in cell wall biogenesis |
|  | NLK58_20285 | *--* | -- |
|  | NLK58_03945 | *exoB* | UDP-glucose 4-epimerase |

**Table S4** Comparison of the genome characteristics of strain CuT 6 with closest *Marinobacter* spp*.*

| Characteristic | *Marinobacter metalresistant* CuT 6 | *Marinobacter Guineae*  M3B^T^ | *Marinobacter profundi*  PSW21^T^ | *Marinobacter*  *lipolyticus*  SM19^T^ |
| --- | --- | --- | --- | --- |
| Size(bp) | 4,411,896 | 4,458,884 | 4,034,607 | 4,020,008 |
| GC content (%) | 57.6 | 57.0 | 60.3 | 56.8 |
| No of contigs genes | 1 | 37 | 39 | 57 |
| Genes for RNA | 59 | 54 | 51 | 53 |
| Total rRNA | 9 | 3 | 3 | 3 |
| AAI | - | 89.31% | 76.61% | 78.78% |
| ANI | - | 88.30% | 76.91% | 77.30% |
| NCBI accession  number | ASM3809864v1 | ASM274473v1 | ASM274471v1 | ASM1842460v1 |

**Table S5** Average nucleotide identity (ANI) and digital DNA-DNA hybridization (dDDH) values between strain CuT 6 and its closest *Marinobacter* relatives

| Strain | **strain CuT 6** | |
| --- | --- | --- |
|  | **ANI (%)** | **DDH (%)** |
| *M. Guineae* M3B^T^ | 88.3% | 43.2±6.5% |
| *M. profundi* PSW21^T^ | 76.9% | 20.0±3.8% |
| *M. lipolyticus* SM19^T^ | 77.3% | 21.7±0.9% |
| *M. gudaonensis* CGMCC 1.6294^T^ | 80.5% | 30.2±6.6% |
| *M. salinus* Hb8^T^ | 78.5% | 25.2±3.4% |
| *M. mobilis* CN46^T^ | 74.4% | 17.3±2.0%, |
| *M. adhaerens* HP15^T^ | 82.3% | 32.9±7.0% |
| *M. segnicrescens* SS011B1–4^T^ | 75.0% | 17.8±3.1% |
| *M. pelagius* HS225^T^ | 78.7% | 24.3±2.3% |
| *M. hydrocarbonoclasticus* ATCC 49840^T^ | 76.5% | 21.4±0.9% |
| *M. salaries* R9SW1^T^ | 76.5% | 20.4±0.5% |

**Table S6** The effects of copper concentration on growth rate variation between *Marinobacter metalliresistant* CuT 6 and its closest *Marinobacter* relatives using ANOVA. *: P>0.05

| **Classification** | **independent variable（Cu^2+^,mM）** | **quadratic sum** | **DF** | **mean square** | ***F*** | ***P*** |
| --- | --- | --- | --- | --- | --- | --- |
| *M. metalresistant* CuT 6-  *M. Guineae* M3B^T^ | 0.0 | 0.006 | 2 | 0.006 | 6.630 | 0.028 |
|  | 0.4 | 0.126 | 2 | 0.126 | 5.597 | 0.040 |
|  | 0.8 | 0.213 | 2 | 0.213 | 5.936 | 0.035 |
|  | 1.2 | 0.056 | 2 | 0.056 | 2.086 | 0.179* |
|  | 1.6 | 0.223 | 2 | 0.223 | 7.284 | 0.022 |
|  | 2.0 | 0 | 2 | 0 | 0.001 | 0.973* |
|  | 4.0 | 1.434 | 2 | 1.434 | 54.929 | 0 |
|  | 6.0 | 0.461 | 2 | 0.461 | 209.991 | 0 |
|  | 8.0 | 0.384 | 2 | 0.384 | 390.324 | 0 |
|  | 10.0 | 0.002 | 2 | 0.002 | 3.123 | 0.108* |
| *M. metalresistant* CuT 6-  *M. profundi* PSW21^T^ | 0.0 | 0.036 | 2 | 0.036 | 5.252 | 0.045 |
|  | 0.4 | 0.119 | 2 | 0.119 | 0.272 | 0.614* |
|  | 0.8 | 0.079 | 2 | 0.079 | 2.108 | 0.177* |
|  | 1.2 | 0.045 | 2 | 0.045 | 1.552 | 0.241* |
|  | 1.6 | 0.060 | 2 | 0.060 | 1.613 | 0.233* |
|  | 2.0 | 0 | 2 | 0 | 0.007 | 0.933* |
|  | 4.0 | 1.383 | 2 | 1.383 | 41.935 | 0 |
|  | 6.0 | 0.529 | 2 | 0.529 | 262.58 | 0 |
|  | 8.0 | 0.296 | 2 | 0.296 | 22.474 | 0.001 |
|  | 10.0 | 0.001 | 2 | 0.001 | 0.194 | 0.669* |

**Fig S1** Growth of highly copper resistant strains, including *Halomonas* sp. CuT 3-1 (A), *Pseudoalteromonas* sp. CuT 4-3 (B) and *Marinobacter metalliresistant* CuT 6 (C). Strains were grown for 85 h at 28°C in MB medium supplemented with the concentration of CuSO_4_ indicated in the legend. Growth was measured by turbidity (OD_600_).

\



**Fig S2** Heavy metal resistance of *Marinobacter* strains, including *Marinobacter metalliresistant* CuT 6, *Marinobacter* sp.CuT 1-2, and other 28 *Marinobacter* type strains deposited in MCCC collection.

Types of added heavy metal compounds: Cu (II): CuSO_4_; Cr (VI): K_2_Cr_2_O_7_; Cd (II): CdCl_2_;

Co (II): CoCl_2_; Zn (II): ZnCl_2_; Hg (II): HgSO_4_

The heavy metal resistance tolerance test uses MB plates containing different concentrations of heavy metal ions to perform streak cultivation on marine bacillus. The presence of colony growth is considered positive, while the absence of colony growth is considered negative. Positive results are confirmed through subculturing. Each experimental result is replicated three times.


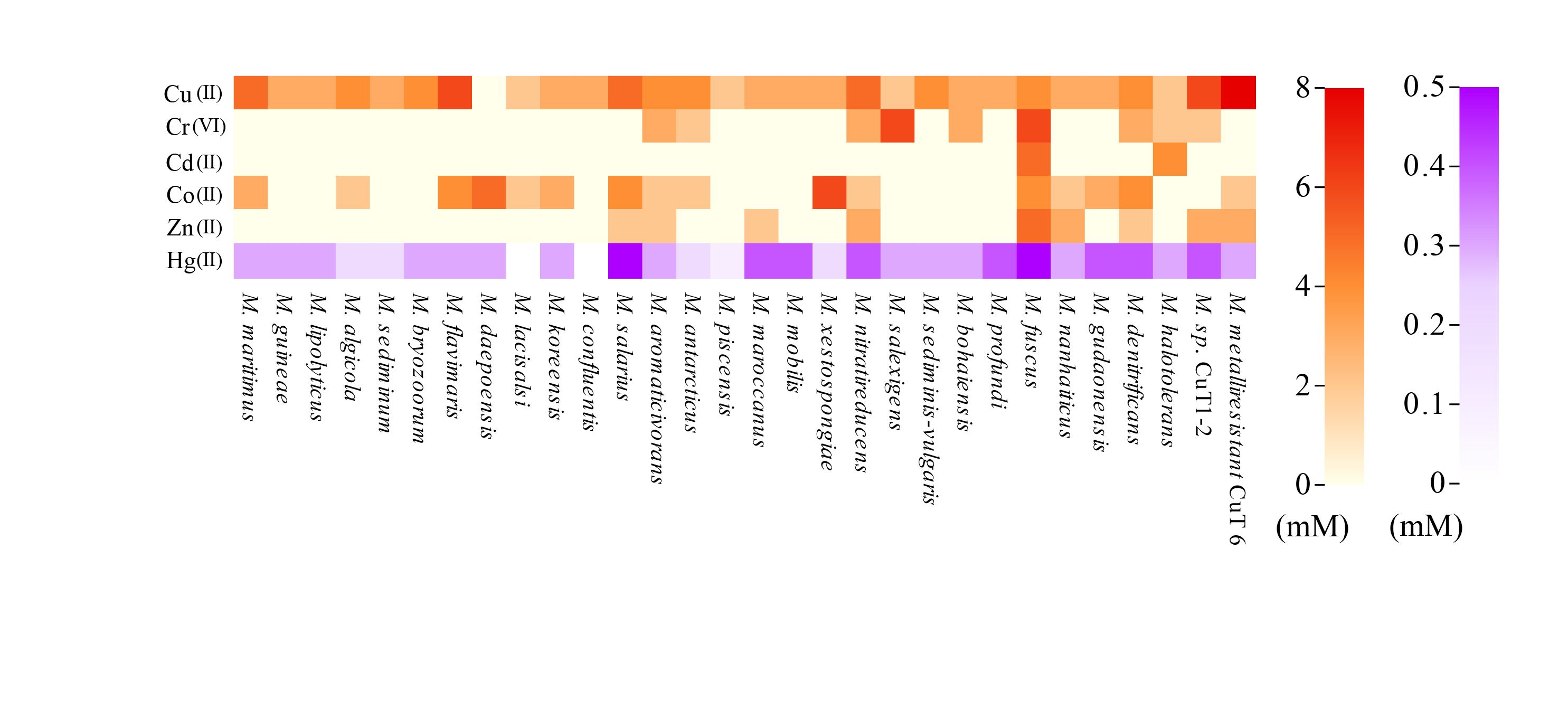


**Fig S3**. The EPA production of the twelve isolates tested by CRA plates and comparison with MB plate cultures.


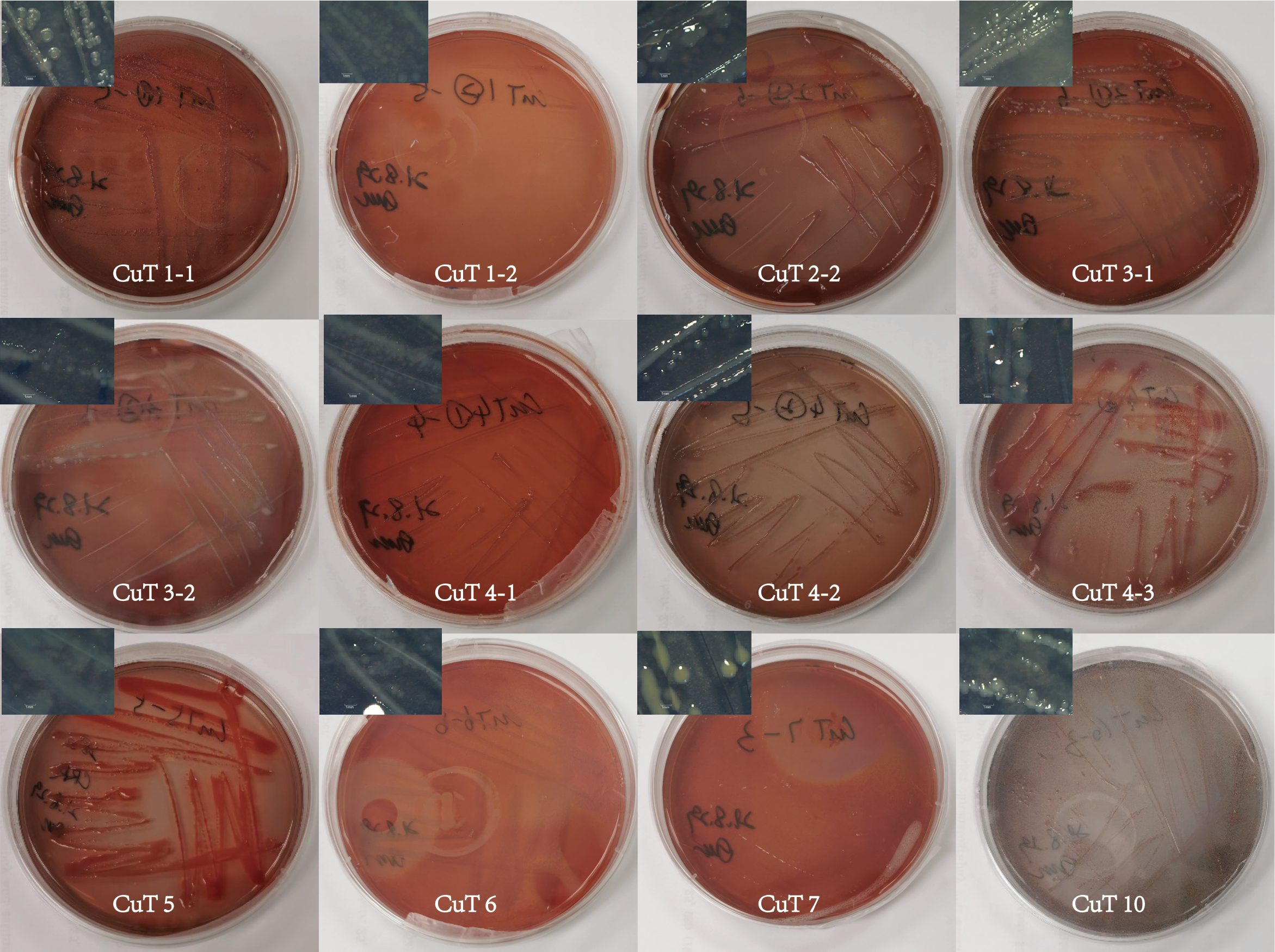


**Fig S4** The contents of polysaccharides and proteins in the crude exopolysaccharide (EPS) produced by *Halomonas* sp. CuT 3-1, *Pseudoalteromonas* sp. CuT 4-3 and *Marinobacter metalliresistant* CuT 6 cultivated under the optimized conditions.


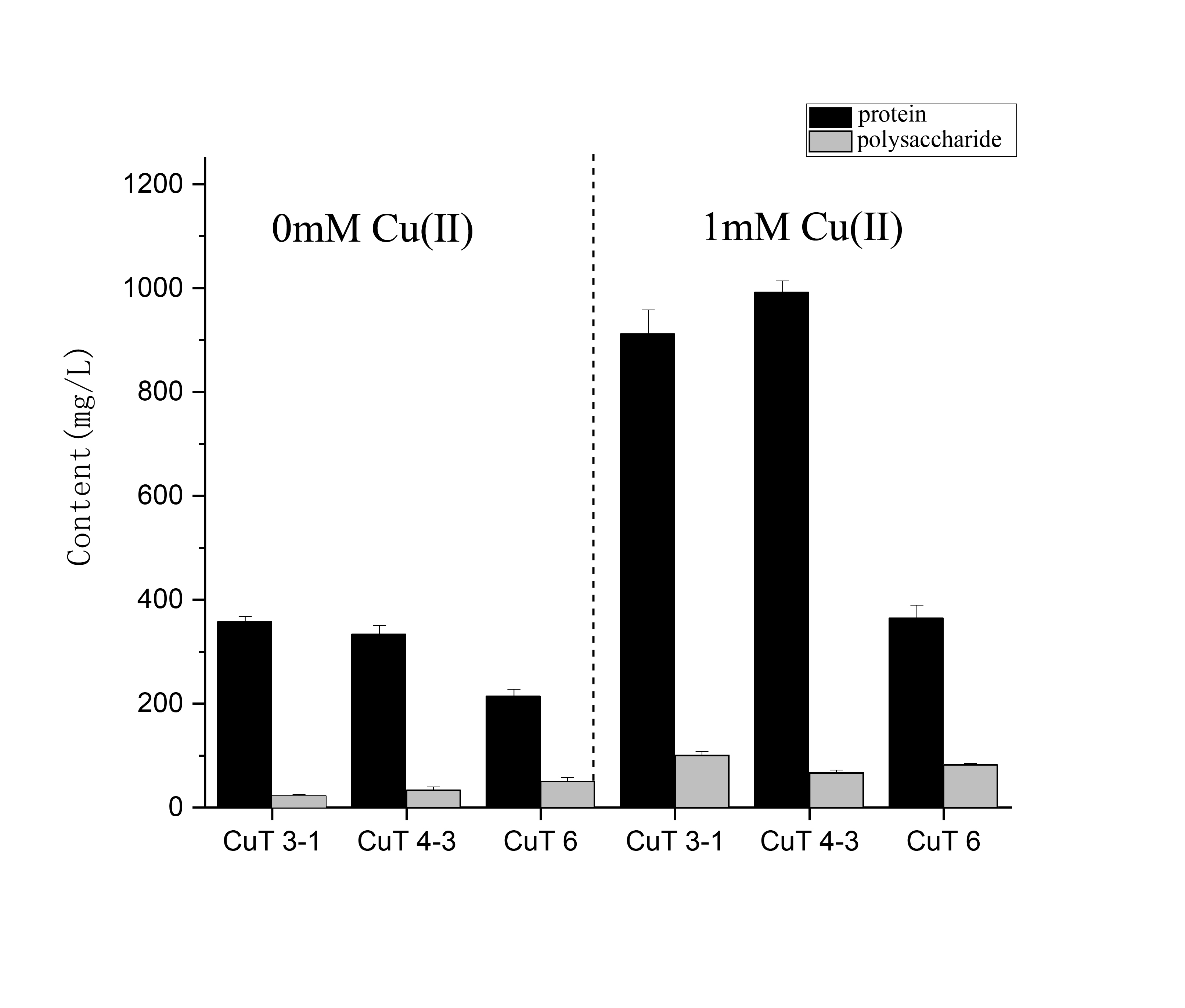


**Fig S5** The copper-resistant strains *Halomonas* sp. CuT 3-1, *Pseudoalteromonas* sp. CuT 4-3, and *Marinobacter metalliresistant* CuT 6 and their adsorption of Cu (II).


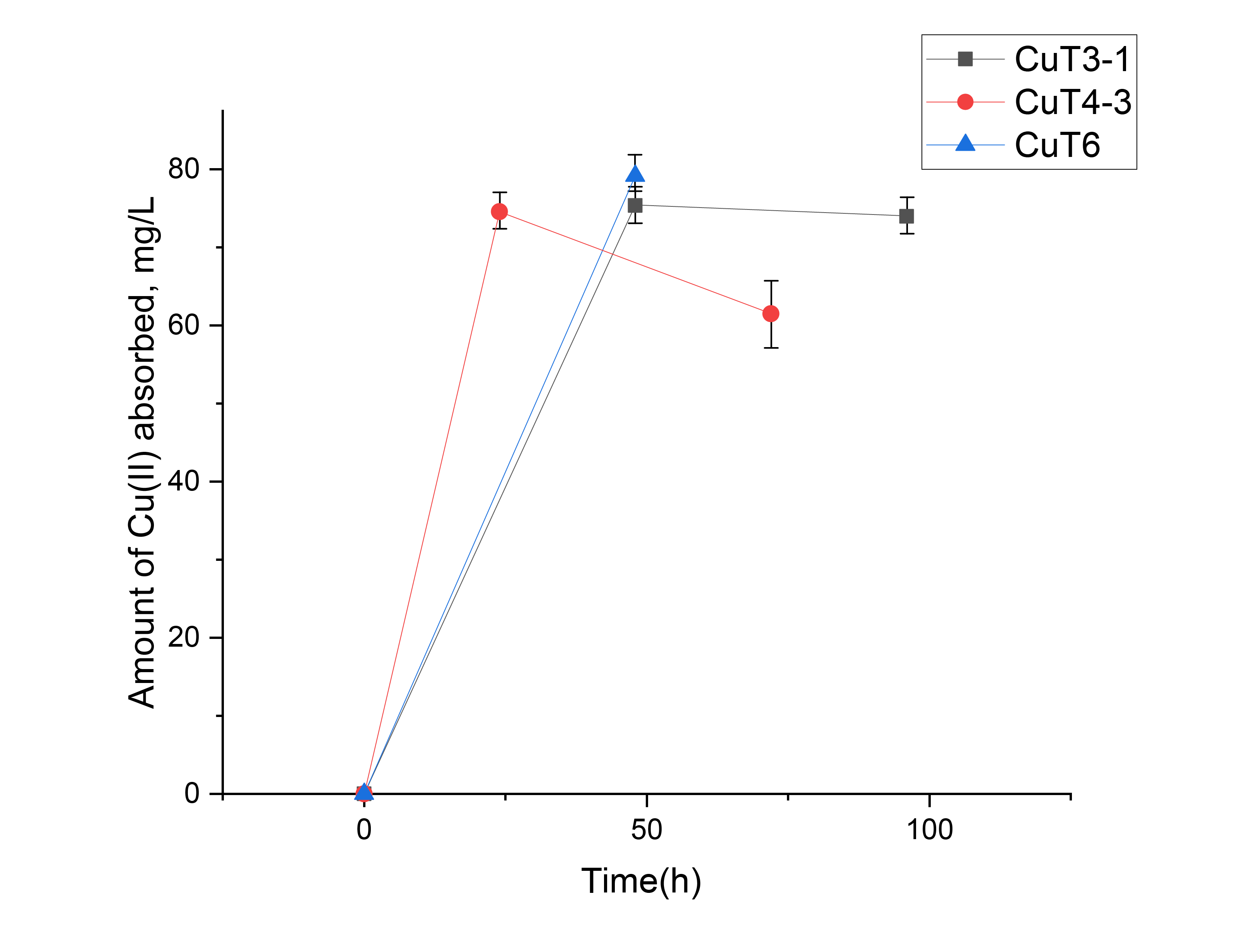


**Fig S6** Phylogenomic tree using the up-to-date bacterial core gene sets (92 genes) containing strain Cut6^T^, the type strains of the phylum *Marinobacter*. *Escherichia coli* in the phylum Escherichia was selected as outgroup. Branch node values below 50 were not shown. Numbers on nodes indicate the gene support index. Bar, 0.10 substitutions per nucleotide position. Numbers in parentheses are sequence accession numbers.


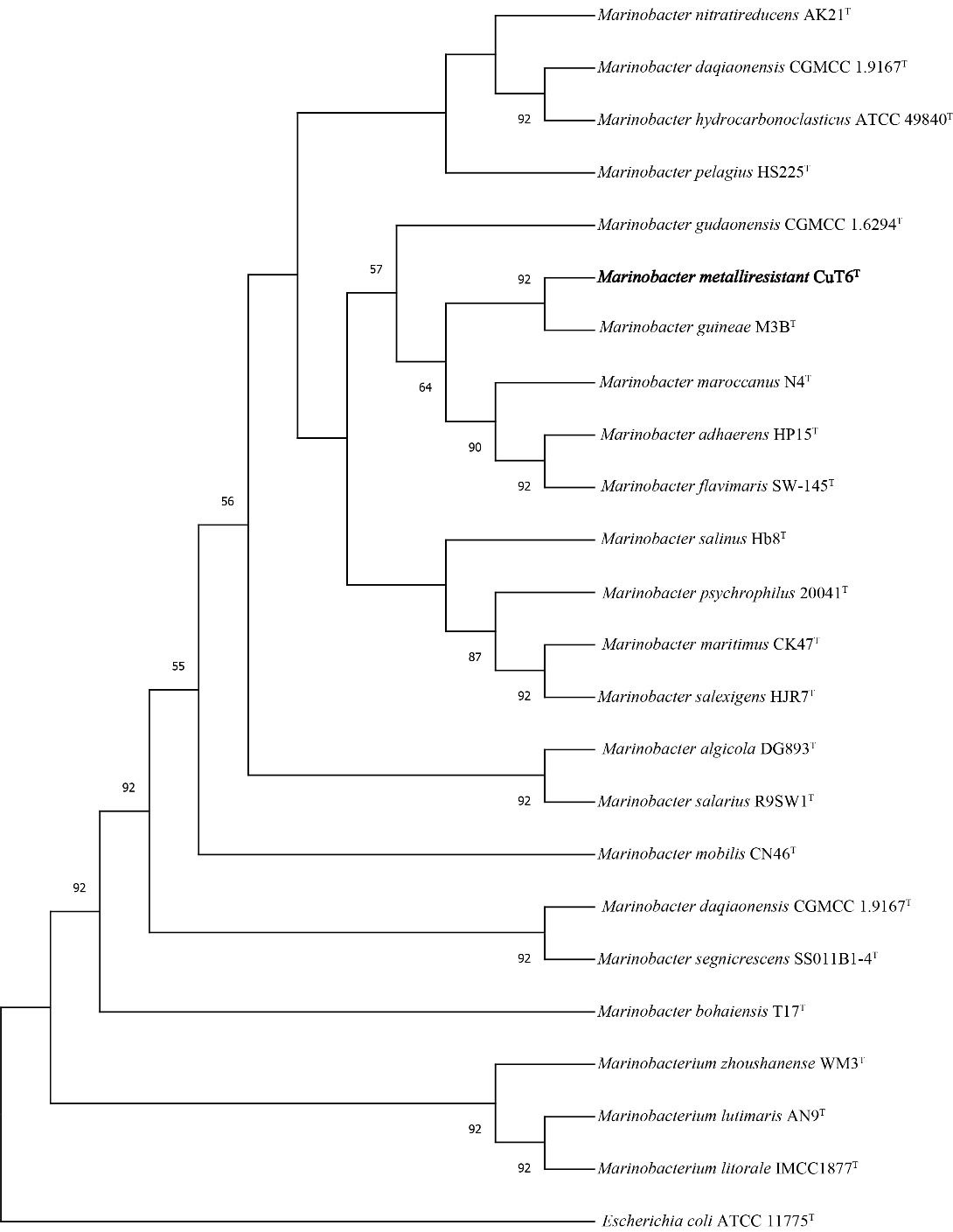


**Fig S7** Electron micrographs of *Marinobacter metalliresistant* CuT 6 grown in the MB medium (A、B) and MB medium with 6mM Cu^2+^(C、D).


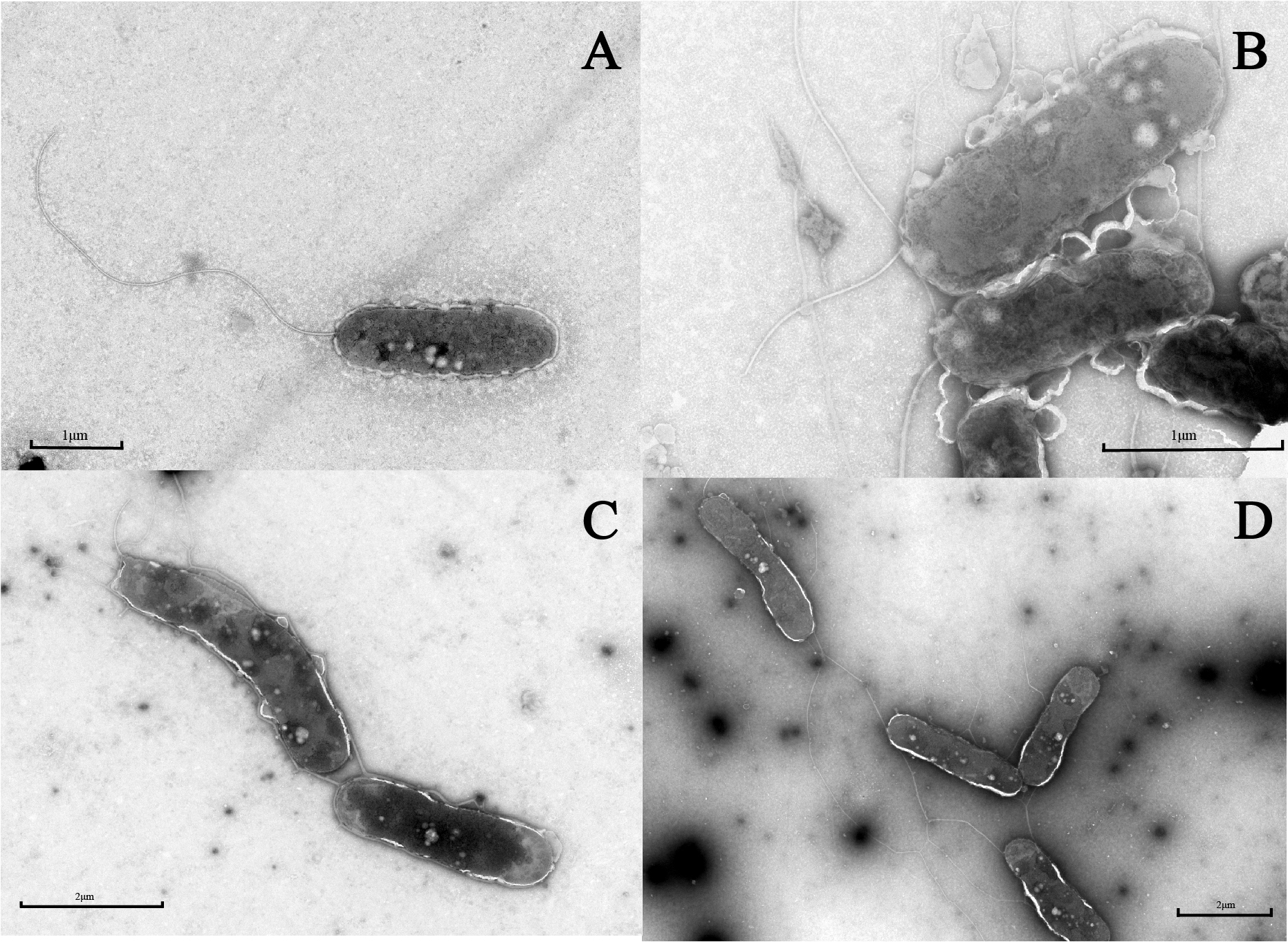


**Fig S8** TLC analysis of polar ester composition of *Marinobacter metalliresistant* CuT6^T^, conducted using Glycolipid (A, staining agent: 1-naphthol sulfate ethanolA), Phospholipid (B, staining agent: molybdenum blue reagent), Total fat: (C, Dyeing agent: Phosphomolybdate ethanol), and Amino ester (D, staining agent: Ninhydrin).

PE: phosphatidylethanolamine; PG:phosphatidylglycerol); GL: diphosphatidylglycerol;

AL: aminolipid; F-first dimension of TLC; S- second dimension of TLC.


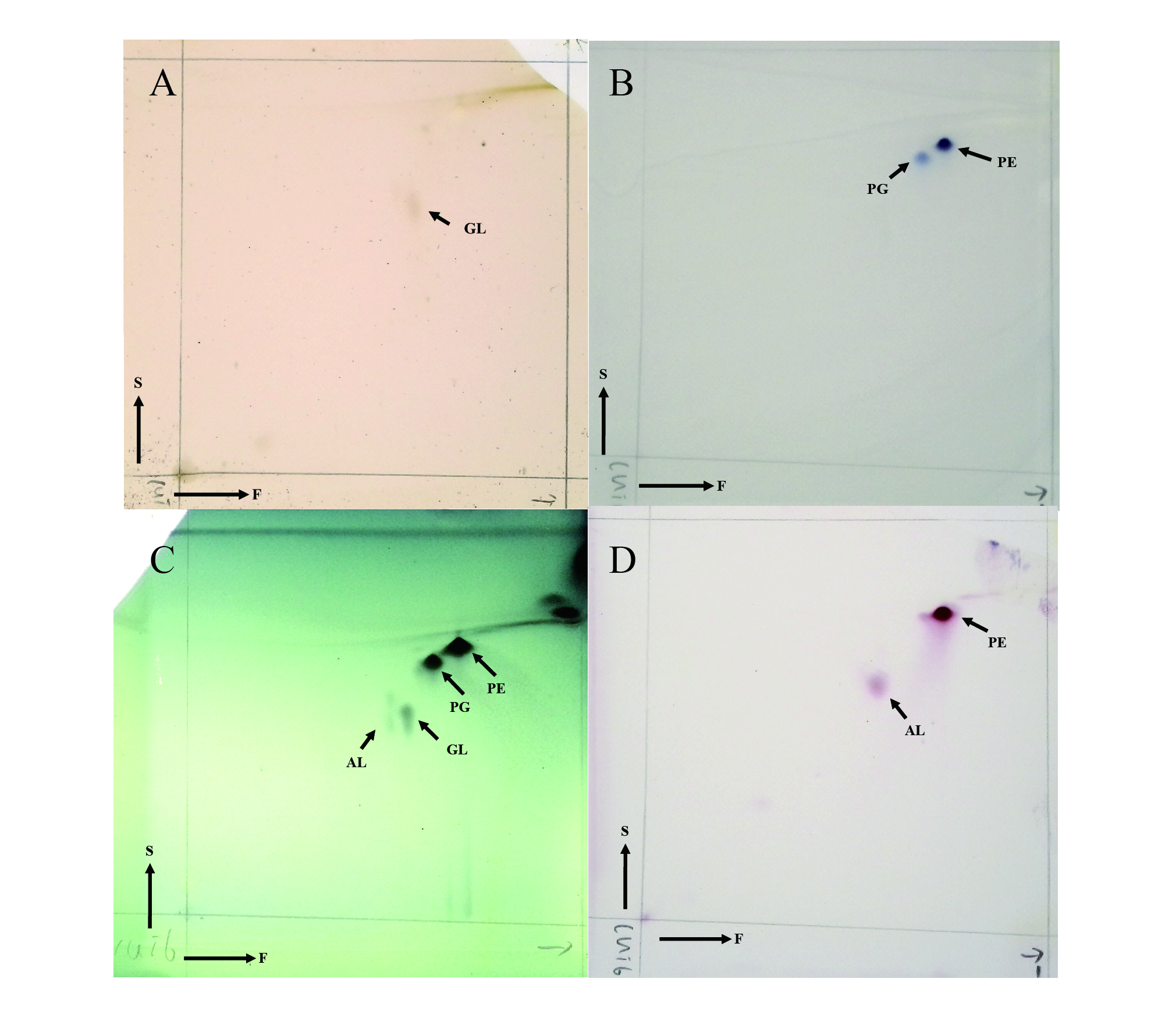


**Fig.S9** Genomic organization of the clusters of heavy metal resistance genes in *Marinobacter metalliresistant* CuT 6.


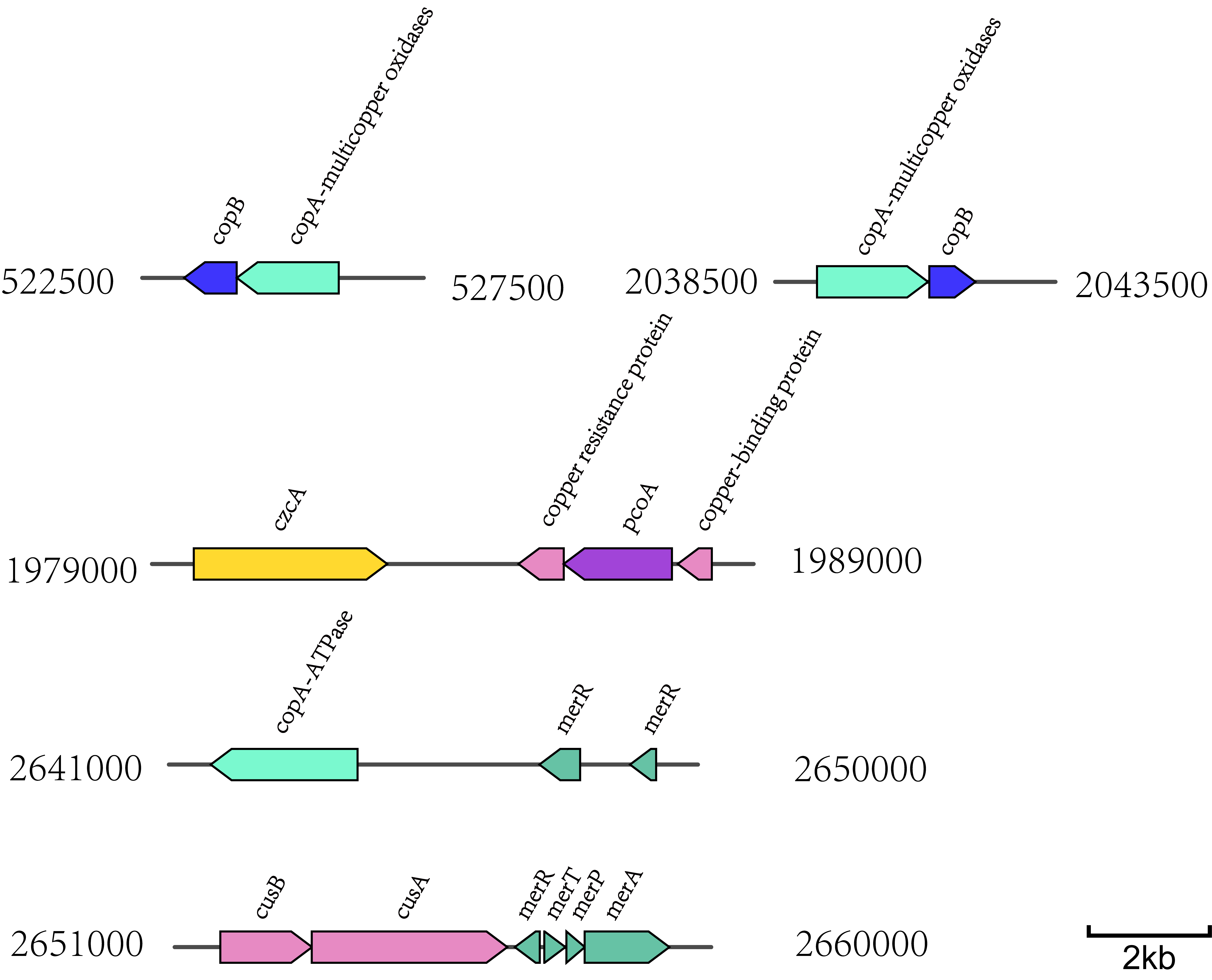


**Fig.S10** The contents of polysaccharides and proteins in the crude exopolysaccharide (EPS) produced by *M.guineae*, *M.flavimaris, M.daepoensis, M.profundi, M.denitrificans* and *M. metalliresistant* CuT 6 cultivated under the optimized conditions


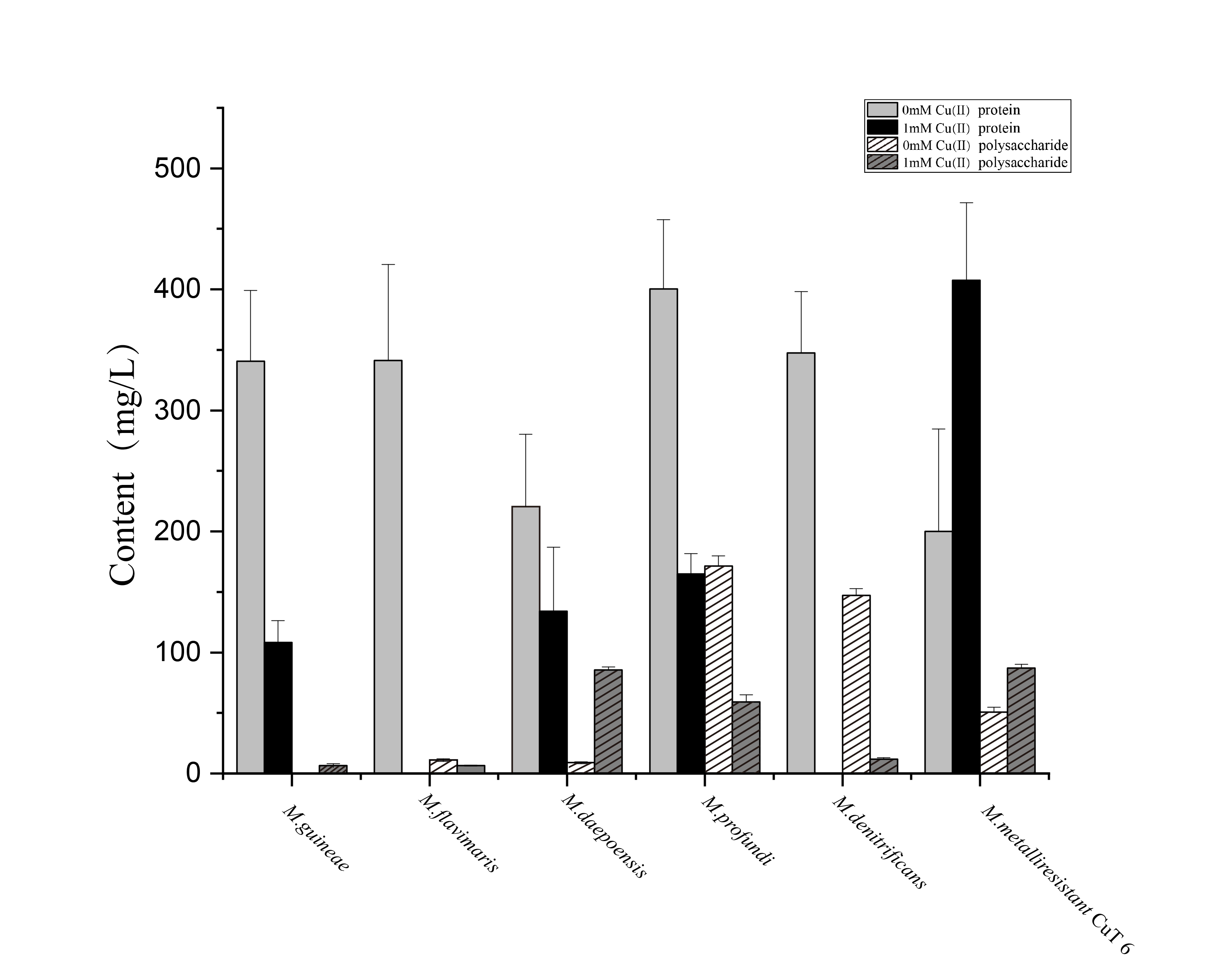

Supplement: Supplementary file 1 [file Data_Sheet_1.docx]
